# Modeling the temporal dynamics of master regulators and CtrA proteolysis in *Caulobacter crescentus* cell cycle

**DOI:** 10.1371/journal.pcbi.1009847

**Published:** 2022-01-28

**Authors:** Chunrui Xu, Henry Hollis, Michelle Dai, Xiangyu Yao, Layne T. Watson, Yang Cao, Minghan Chen

**Affiliations:** 1 Genetics, Bioinformatics, and Computational Biology, Virginia Tech, Blacksburg, Virginia, United States of America; 2 Department of Computer Science, Wake Forest University, Winston-Salem, North Carolina, United States of America; 3 Department of Computer Science, Virginia Tech, Blacksburg, Virginia, United States of America; Pázmány Péter Catholic University: Pazmany Peter Katolikus Egyetem, HUNGARY

## Abstract

The cell cycle of *Caulobacter crescentus* involves the polar morphogenesis and an asymmetric cell division driven by precise interactions and regulations of proteins, which makes *Caulobacter* an ideal model organism for investigating bacterial cell development and differentiation. The abundance of molecular data accumulated on *Caulobacter* motivates system biologists to analyze the complex regulatory network of cell cycle via quantitative modeling. In this paper, We propose a comprehensive model to accurately characterize the underlying mechanisms of cell cycle regulation based on the study of: **a)** chromosome replication and methylation; **b)** interactive pathways of five master regulatory proteins including DnaA, GcrA, CcrM, CtrA, and SciP, as well as novel consideration of their corresponding mRNAs; **c)** cell cycle-dependent proteolysis of CtrA through hierarchical protease complexes. The temporal dynamics of our simulation results are able to closely replicate an extensive set of experimental observations and capture the main phenotype of seven mutant strains of *Caulobacter crescentus*. Collectively, the proposed model can be used to predict phenotypes of other mutant cases, especially for nonviable strains which are hard to cultivate and observe. Moreover, the module of cyclic proteolysis is an efficient tool to study the metabolism of proteins with similar mechanisms.

## Author summary

Timed cellular events in both eukaryotes and prokaryotes, such as chromosome replication, transcription, cell differentiation, cytokinesis, and cell division, are controlled by remarkably complex genetic regulations and protein-protein interactions. In this work, we investigate the cell cycle of *Caulobacter crescentus*, an alphaproteobacterium undergoing asymmetric cell divisions, to understand mechanisms underlying temporal regulations of complex cellular events. The asymmetric lifestyle makes *Caulobacter crescentus* easily synchronized and tracked, which is the foundation of molecular data accumulation. Here, we utilize the mathematical modeling together with experimental information to systematically integrate the complex gene-protein and protein-protein interactions in cell cycle progression. Using the mathematical model, we capture core features of cell cycle-dependent methylation, transcription, and proteolysis. In mutant cases, we found the complex and redundant regulatory network ensure the robustness of *Caulobacter crescentus* system because the change of most molecules does not cause immediate mortality, although they influence the time points of cell differentiation and division. The overall model and individual modules such as simulating transcriptional regulations and protease complexes can be further extended to the study of cell development in other bacterial species.

## Introduction

**Fig 1:**
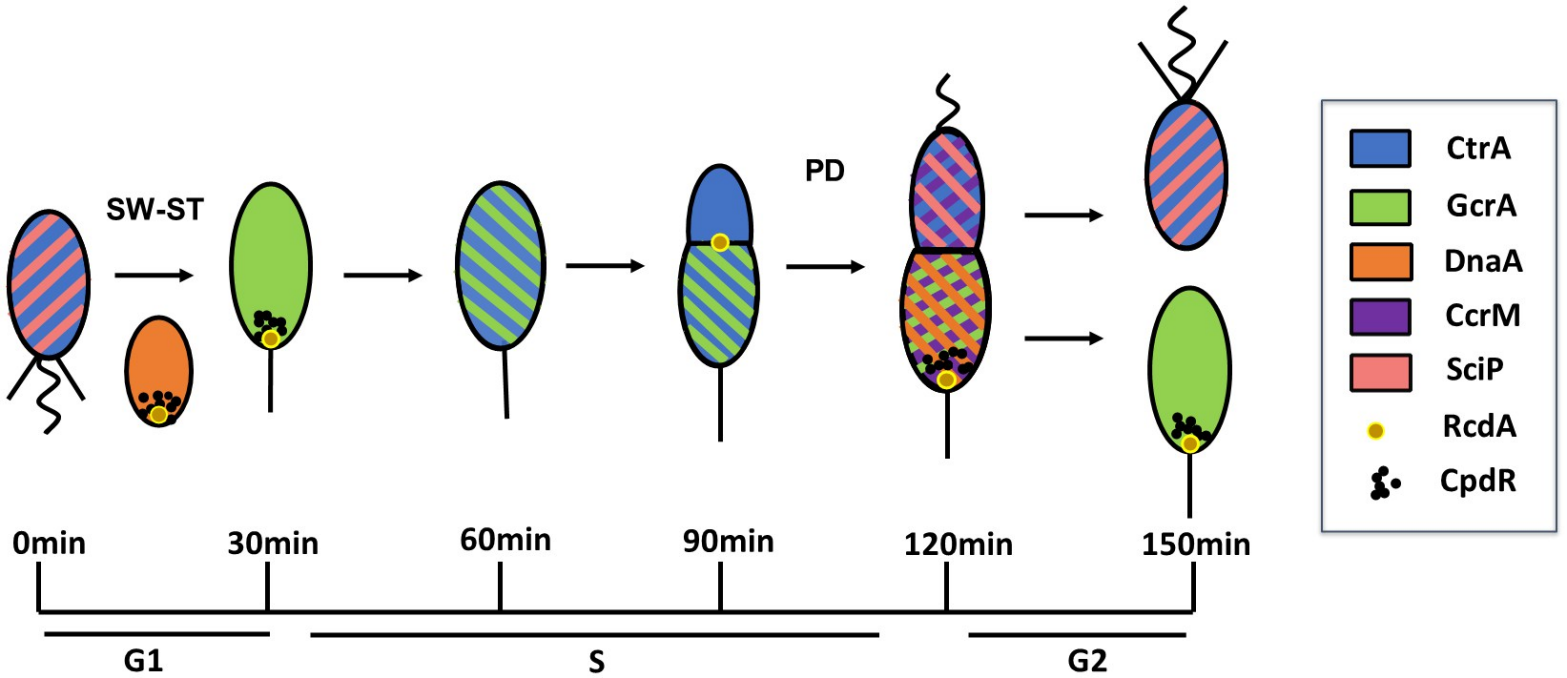
The asymmetric cell cycle of *C. crescentus* including G1, S, and G2 phases. *C. crescentus* cell grows in G1, replicates DNA in S phase, and prepares for cell division in G2 phase. The predivisional (PD) cell divides asymmetrically into two different progenies: motile swarmer (SW) cell and non-motile stalked cell (ST). The dynamics of CtrA, GcrA, DnaA, CcrM, SciP, RcdA, and CpdR is indicated by color during each stage of the cell cycle.

*Caulobacter crescentus* (*C. crescentus*) is a model organism for exploring cell development and cell cycle regulation in prokaryotes. *C. crescentus* undergoes an asymmetric cell division producing two distinct progenies: a sessile stalked cell equipped with a stalk and a motile swarmer cell equipped with a flagellum (Fig 1). While the stalked cell immediately initiates chromosome replication and enters the next cell cycle, the swarmer cell searches for suitable environments and differentiates into a stalked cell (sw-to-st transition) before entering the cell cycle replication [1]. The dimorphic lifestyle makes *C. crescentus* feasible to survive in oligotrophic waters.

**Fig 2:**
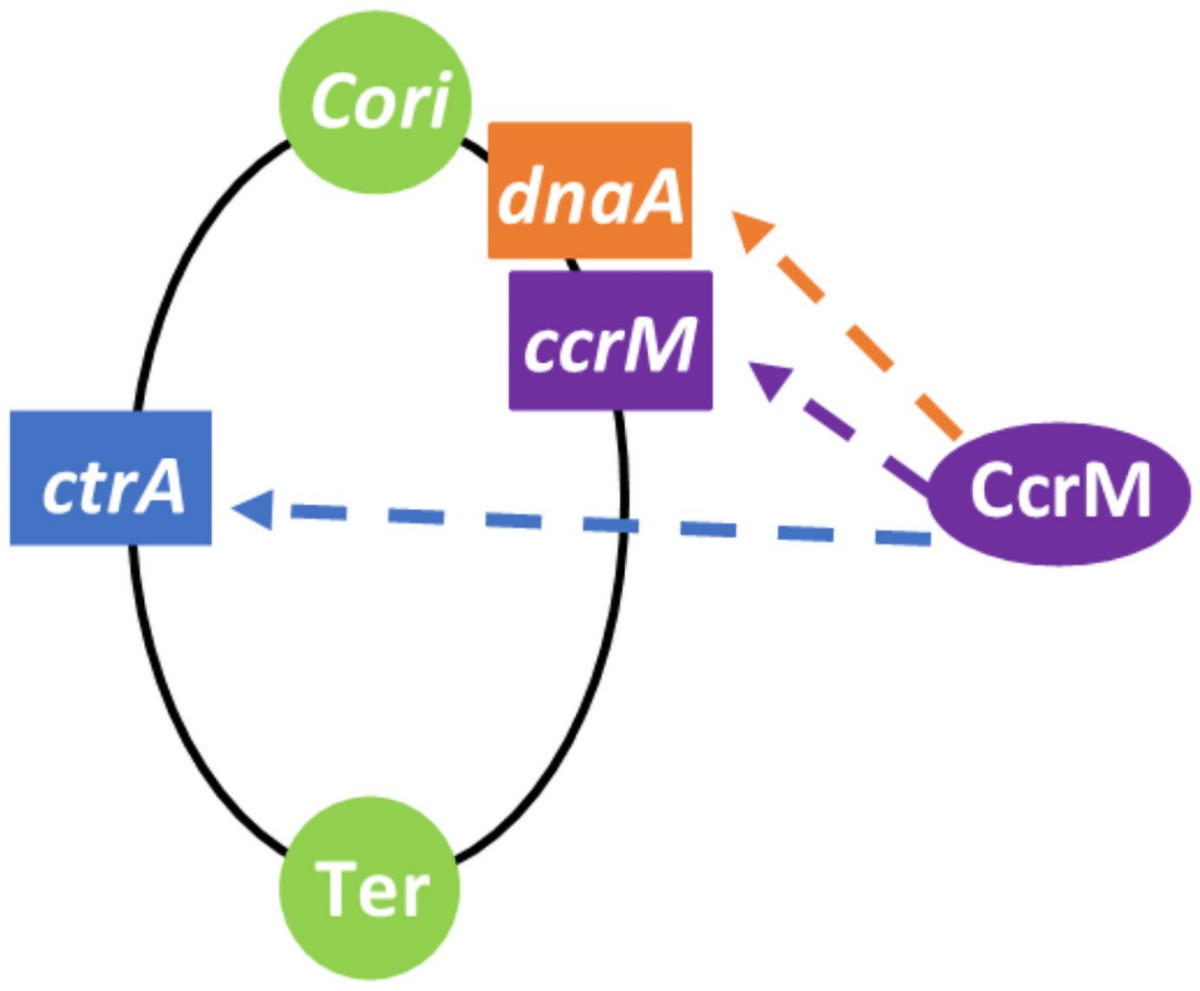
Methylation site locations of different genes on *C. crescentus* chromosome. The elliptical curve represents the DNA fork in replication. *Cori* is the origin of DNA replication and *Ter* is the termination site. The CcrM methylation site is located upstream of the *dnaA*, *ccrM* and *ctrA* genes, represented as rectangles.

The timed asymmetric cell progression of *C. crescentus* is highly regulated by a cell cycle-dependent regulatory network including four master regulators–DnaA, GcrA, CtrA, and CcrM [2, 3]. DnaA, GcrA, and CtrA are transcriptional factors that control over 200 cell cycle-regulated genes in *C. crescentus*. These proteins form a loop to control each other. DnaA activates *gcrA* expression, GcrA regulates the expression of *ctrA* and *dnaA*, and CtrA in turn influences the transcription of *dnaA* [4–6]. Furthermore, DnaA is a conserved DNA replication initiator, activating replication by binding directly with the chromosome origin (*Cori*) [7]. In addition, there are five binding sites for CtrA on *Cori*, where replication initiation is suppressed when being bound by the phosphorylated form of CtrA (CtrA∼P) [4]. CcrM, a conserved methyltransferase, is turned on at the completion of DNA replication to fully methylate the motif GANTC, which is carried by promoters of *ctrA*, *dnaA*, and *ccrM* (see Fig 2) [8, 9]. The short window of CcrM allows the maintenance of hemimethylated chromosome during replication, ensuring the robustness of cell cycle development. Moreover, CcrM has been reported to influence the expressions of more than 10% genes [8, 10]. Among these CcrM-regulated genes, more than 100 genes are likely influenced by a GANTC motif-dependent pathways, while the mechanisms of the rest genes are not clear [8]. Here, we take the CcrM-dependent methylation of GANTC motif into the regulatory network although it is dispensable for the replication control. Additionally, SciP is an antagonist of CtrA which is instrumental in cell cycle regulations but receives little attention. SciP spatiotemporally represses the transcription of CtrA-induced genes because most of these genes contain a SciP binding site upstream of a CtrA binding site in their promoters [2].

A wealth of experimental data for cell cycle-regulated genes and proteins in *C. crescentus* have been accumulated in last decade [11, 12]. System biologists have proposed different quantitative models to analyze underlying mechanisms and pathways of cell cycle regulation. For example, Li *et al*. [4, 13] quantitatively modeled the interactions between CtrA, DnaA, GcrA, and CcrM and studied the simulated behaviors of some mutants. Murray *et al*. [10] proposed a simplified model incorporating CtrA, CckA, and GcrA to capture the cell cycle features of *C. crescentus* and predict the behaviors of Δ*gcrA* cells. However, the proteolysis of CtrA is not explicitly modeled. Li *et al*. [4, 13] borrowed DivK while Murray *et al*. [10] used CckA to describe the proteolysis of CtrA; but both DivK and CckA are indirect factors influencing the proteolysis of CtrA through phosphorelay pathways [14]. There are a series of models working on the spatial regulatory networks in *C. crescentus*. Li et al. investigated the spatial regulations focusing on CtrA in a stochastic model [15], which preliminarily revealed roles of spatial phosphorylation on the asymmetric cell cycle in *C. crescentus*. Further, Chen et al. [16] and Xu et al. [17] proposed spatial models for the scaffolding protein PopZ in *C. crescentus*, which complemented Li et al.’s model [15] about the initial localization factors. Although previous mathematical models revealed some mechanisms of *Caulobacter* system, a comprehensive model for core regulators of the cell development, as well as a quantitative comparison between simulations and observations, have yet been explicitly investigated. Previous models didn’t consider the mRNA abundance and transcription process based on master regulators. Additionally, there is no mathematical model describing the cyclic proteolysis of master regulator CtrA, which plays important roles for cell development especially for the sw-to-st transition [18].

In this paper, we focus on five core components–DnaA, GcrA, CtrA, CcrM, and SciP that control over 90% of cell cycle-regulated genes, and propose a mathematical model that considers the regulation of DNA replication and methylation, as well as the gene-protein and protein-protein interactions. Since CtrA is essential in the cell cycle regulation and its proteolysis is distinctively and spatiotemporally regulated, we construct a hierarchical ClpXP complex network for its proteolysis, which is then integrated into the entire model. The simulated dynamics of mRNA and proteins are consistent with experimental observations. The ClpXP complex model can be used as a quantitative analysis tool to simulate other cyclic proteolysis in *C. crescentus*, such as the proteolysis of ShkA and TacA [19, 20].

## Materials and methods

### Model description

The regulatory network of bacterial cell cycle includes a series of complex mechanisms, such as genetic regulations, degradations, phosphorylation, dephosphorylation, and so on. The details of the complex regulatory network will be described in the following.

#### Module 1: The core regulatory network of cell cycle

**Fig 3:**
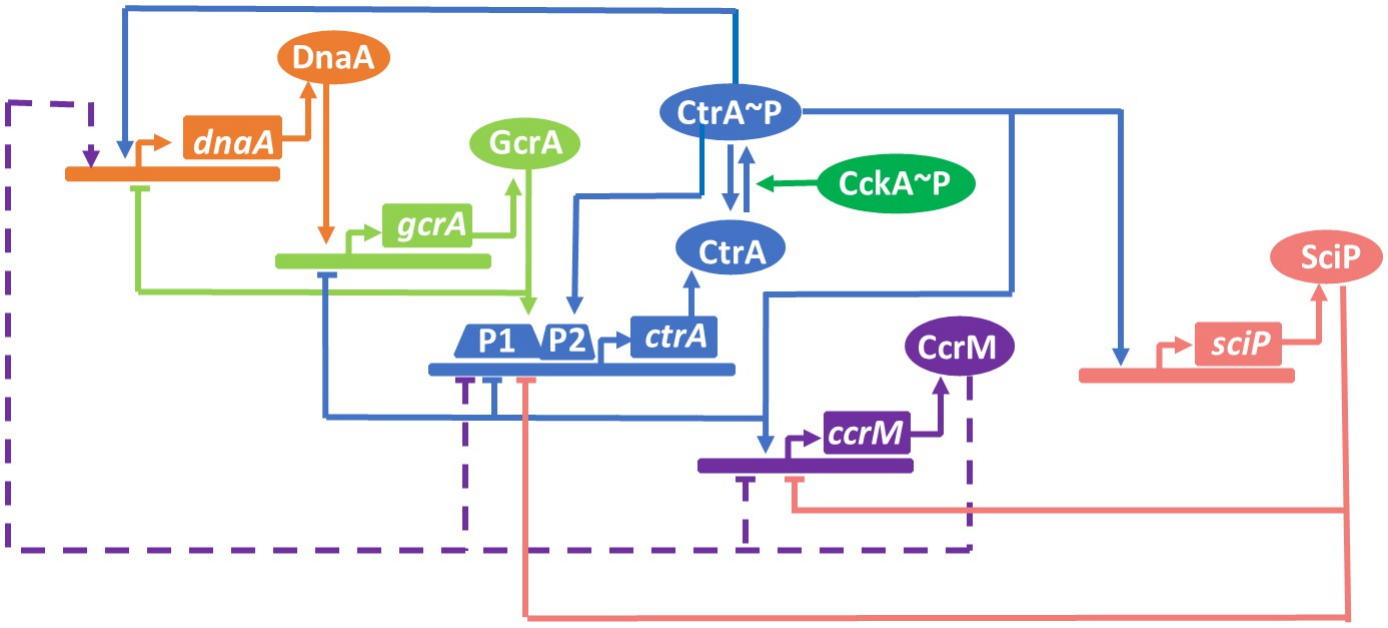
The master regulatory network of *C. crescentus*. Solid lines represent activation/inhibition influences of master regulators (DnaA, GcrA, CcrM, CtrA, SciP) with arrow/bar, respectively. The dashed lines represent the methylation effects on *dnaA*, *ctrA*, *ccrM* genes from CcrM.

The master regulatory network of *C. crescentus*, as summarized in Fig 3, is composed of DnaA (*dnaA*), GcrA (*gcrA*), CtrA (*ctrA*), CcrM (*ccrM*), and SciP (*sciP*). Specifically, DnaA promotes the expression of *gcrA*, while GcrA inhibits DnaA and activates one of the promoters (P1) of CtrA [7]. Conversely, CtrA∼P suppresses the initiation of DNA replication [21], activates the transcription of *dnaA* [4], inhibits the activity of P1, and activates itself through another promoter (P2) [3]. The accumulation of CtrA promotes the expressions of *ccrM* and *sciP*, where CcrM controls the methylation state of P1 of *ctrA* [22]. SciP downregulates CtrA and CcrM [2]. The regulatory network of the five master proteins and mRNAs governs cell cycle-regulated genes, thereby driving the cell cycle progression [11].

In normal cell cycle progression, active CtrA (phosphorylated form, blue color in Fig 1) is cleared during the sw-to-st transition; CtrA concentrations are generally low in stalked cells when the Z-ring is closed [23]. The activity of CtrA is controlled by synthesis, degradation, and phosphorylation, the latter of which is driven by the CckA-dependent pathway [18, 24] (Fig 3). As CckA∼P is the only known phosphoryl donor of CtrA [14], we involve the CckA-dependent phosphotransfer into our model. CtrA proteolysis depends on a particular protease complex comprising the protease ClpXP and four additional adaptors–CpdR, RcdA, PopA, and c-di-GMP (cdG) [18]. While the protease ClpXP presents throughout the entire cell cycle, RcdA and CpdR co-localize at the stalked pole during sw-to-st transition and stay in the predivisional cell’s stalked compartment (gold and black circles in Fig 1) [25]. The phosphorylation of CpdR is also regulated by CckA, thus CckA regulates the activity of CtrA through both phosphorylation and proteolysis. Additionally, CtrA controls the expression of RcdA and CpdR in *C. crescentus*.

#### Module 2: Cell cycle-dependent proteolysis of CtrA

**Fig 4:**
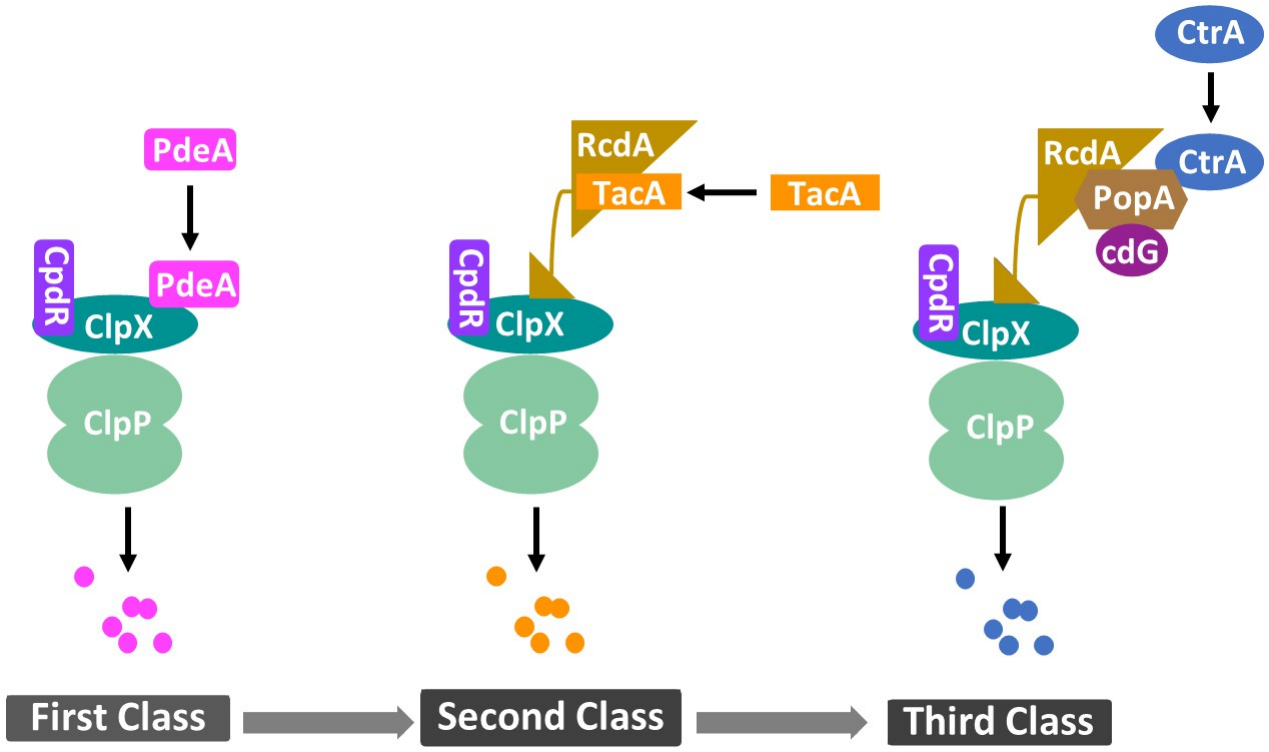
Hierarchical proteolysis of the first (eg. PdeA), second (eg. TacA), and third (eg. CtrA) substrate. The degradation of different substrates is dependent on the degree of adaptor assembly. Priming of the protease ClpXP by unphosphorylated CpdR results in PdeA decay, which recruits additional adaptor RcdA to degrade TacA. RcdA tethers cdG-bound PopA with primed ClpXP, which is responsible for the proteolysis of CtrA.

The stability and activity of proteins strictly regulate cell cycle processes. Accordingly, proteolysis plays a significant role in cell development and response to internal/external stimuli [18, 26]. ClpXP, a highly conserved protease, is responsible for the proteolysis of a wide range of proteins including CtrA in *C. crescentus* [18]. Many substrates of ClpXP are cell cycle-regulated. Although ClpXP levels do not change significantly throughout the cell cycle, it requires additional cell cycle-dependent adaptors to cyclically degrade proteins [18]. Substrates of ClpXP-based proteolysis require different classes of protease complex assemblies [27]. Substrates, such as PdeA, only require ClpXP primed by unphosphorylated CpdR; we name this type of substrates as the first class substrate. Similarly, the second class substrates require primed ClpXP additionally with RcdA assembled; and the third class substrates, such as CtrA, require binding between PopA and c-di-GMP connected with the second class protease complex (see Fig 4).

**Fig 5:**
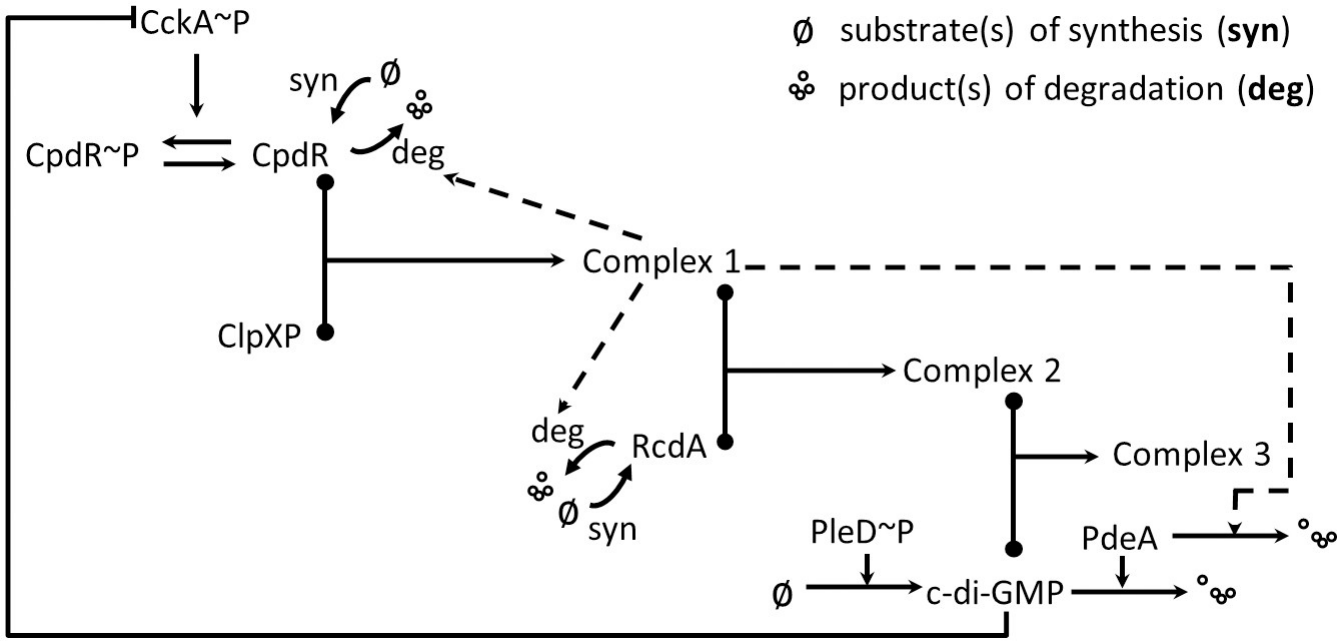
Hierarchical diagram of protease complexes. Solid lines with arrow denote metabolisms; solid lines with filled circles denote binding processes; dashed lines with arrow denote activation effects. Complex 1 decays the first class of substrates; Complex 2 degrades the second class of substrates; Complex 3 degrades the third class of substrates.

To simulate the protease complex for CtrA degradation, we use ‘Complex 1’, ‘Complex 2’, and ‘Complex 3’ to name the protease complexes that degrade the first, second, and third class substrates, respectively (see Fig 5). Unphosphorylated CpdR primes ClpXP to function as the first class protease complex (Complex 1) which degrades CpdR in turn [14]. (Table 1, Eq. 20). Primed ClpXP (Complex 1) recruits RcdA (Complex 2) to deliver the second class substrates to the protease ClpXP [27] (Table 1, Eq. 23). Additionally, the RcdA proteolysis has been shown to be catalyzed by Complex 1 [20]. Besides CpdR and RcdA, the third class proteolysis requires PopA bound with cdG, where cdG-bound PopA directly interacts with RcdA and CtrA ensuring the specific degradation of CtrA [18]. The diguanylate cyclase PleD and phosphodiesterase PdeA are included in our system as the main synthetase and hydrolase of cdG, respectively, where the expression of *pleD* and *pdeA* is controlled by CtrA∼P [28]. PdeA is proteolyzed by Complex 1, shown in Fig 5. As PopA bears a GGDEF domain and two receiver domains akin to PleD, we assume PopA functions as a dimer; thus, PopA dimer binds with two cdG molecules in the same way as PleD does [29, 30]. Since cdG levels in *C. crescentus* are less than 0.3 *μ*M [31], which is much lower than most protein levels, we use cdG to represent the PopA:2c-di-GMP binding species in this model (Fig 5). Moreover, the phosphorylation of CpdR is controlled by the kinase CckA, similarly with CtrA [14]. cdG binds to CckA to inhibit its kinase activity [32], which means cdG participates in the degradation and dephosphorylation of CtrA. CckA and cdG connect the master regulators network and ClpXP-based proteolysis system through CtrA (Fig 5).

**Table 1:** Equations of replication and methylation, transcription, translation, and proteolysis.

	**Equations of DNA**
(1)	$\frac{dIni}{dt}=k_{\text{s},\text{Ini}}\cdot \frac{{\left(\frac{\left[\text{DnaA}\right]}{\Theta_{\text{DnaA}}}\right)}^{4}}{J_{\text{a},\text{Ini}}^{4}+{\left(\frac{\left[\text{CtrA}∼\text{P}\right]}{\Theta_{\text{ctrA}}}\right)}^{4}+{\left(\frac{\left[\text{DnaA}\right]}{\Theta_{\text{DnaA}}}\right)}^{4}}\cdot (1+\frac{1}{J_{\text{i},\text{Ini}}^{4}+{\left(\frac{h_{Cori}}{\Theta_{\text{Cori}}}\right)}^{4}})$
(2)	$\frac{dElong}{dt}=k_{\text{elong}}\cdot \frac{Elong^{4}}{Elong^{4}+P_{\text{elong}}^{4}};\;\text{(3)}\;\frac{dZring}{dt}=k_{s,Zring}$
(4)	$\frac{dh_{Cori}}{dt}=-k_{\text{m},Cori}\cdot \frac{{\left[\text{CcrM}\right]}^{4}}{J_{\text{m},Cori}^{4}+{\left[\text{CcrM}\right]}^{4}}\cdot h_{Cori}$
(5)	$\frac{dh_{ccrM}}{dt}=-k_{\text{m},ccrM}\cdot \frac{{\left[\text{CcrM}\right]}^{4}}{J_{\text{m},ccrM}^{4}+{\left[\text{CcrM}\right]}^{4}}\cdot h_{ccrM}$
(6)	$\frac{dh_{ctrA}}{dt}=-k_{\text{m},ctrA}\cdot \frac{{\left[\text{CcrM}\right]}^{4}}{J_{\text{m},ctrA}^{4}+{\left[\text{CcrM}\right]}^{4}}\cdot h_{ctrA}$
	**Equations of mRNAs**
(7)	$\frac{dI_{ccrM}}{dt}=k_{\text{s},I_{ccrM}}\cdot (\frac{[\text{CtrA}∼{\text{P]}}^{2}}{J_{\text{a},ccrM\text{-CtrA}}^{2}+[\text{CtrA}∼{\text{P]}}^{2}}\cdot \frac{J_{\text{i},ccrM\text{-SciP}}^{2}}{J_{\text{i},ccrM\text{-SciP}}^{2}+{\left[\text{SciP}\right]}^{2}})\cdot h_{ccrM}-k_{\text{d},I_{ccrM}}\cdot I_{ccrM}$
(8)	$\frac{dccrM}{dt}=k_{\text{s},ccrM}\cdot I_{ccrM}-k_{\text{d},ccrM}\cdot ccrM$
(9)	$\frac{ddnaA}{dt}=k_{\text{s},dnaA}\cdot (\frac{J_{\text{i},dnaA\text{-GcrA}}^{2}}{J_{\text{i},dnaA\text{-GcrA}}^{2}+{\left[\text{GcrA}\right]}^{2}})\cdot \left(2-h_{Cori}\right)-k_{\text{d},dnaA}\cdot dnaA$
(10)	$\frac{dgcrA}{dt}=k_{\text{s},gcrA}\cdot (\frac{{\left[\text{DnaA}\right]}^{2}}{J_{\text{a},gcrA\text{-DnaA}}^{2}+{\left[\text{DnaA}\right]}^{2}}\cdot \frac{J_{\text{i},gcrA\text{-CtrA}}^{2}}{J_{\text{i},gcrA\text{-CtrA}}^{2}+[\text{CtrA}∼{\text{P]}}^{2}})-k_{\text{d},gcrA}\cdot gcrA$
(11)	$\frac{dsciP}{dt}=k_{\text{s},sciP}\cdot \frac{[\text{CtrA}∼{\text{P]}}^{2}}{J_{\text{a},sciP\text{-CtrA}}^{2}+[\text{CtrA}∼{\text{P]}}^{2}}-k_{\text{d},sciP}\cdot sciP$
(12)	$\frac{dctrA}{dt}=k_{\text{s1},ctrA}\cdot (\frac{{\left[\text{GcrA}\right]}^{2}}{J_{\text{a},ctrA\text{-GcrA}}^{2}+{\left[\text{GcrA}\right]}^{2}}\cdot \frac{J_{\text{i},ctrA\text{-CtrA}}^{4}}{J_{\text{i},ctrA\text{-CtrA}}^{4}+[\text{CtrA}∼{\text{P]}}^{4}}\cdot \frac{J_{\text{i},ctrA\text{-SciP}}^{4}}{J_{\text{i},ctrA\text{-SciP}}^{4}+{\left[\text{SciP}\right]}^{4}})\cdot h_{ctrA}+k_{\text{s2},ctrA}\cdot \frac{{\left[\text{CtrA}∼\text{P}\right]}^{2}}{J_{\text{a},ctrA\text{-CtrA}}^{2}+[\text{CtrA}∼{\text{P]}}^{2}}-k_{\text{d},ctrA}\cdot ctrA$
	**Equations of regulatory proteins**
(13)	$\frac{d\left[\text{CcrM}\right]}{dt}=k_{\text{s},\text{CcrM}}\cdot ccrM-k_{\text{d},\text{CcrM}}\cdot \left[\text{CcrM}\right]$
(14)	$\frac{d\left[\text{DnaA}\right]}{dt}=k_{\text{s},\text{DnaA}}\cdot dnaA-k_{\text{d},\text{DnaA}}\cdot \left[\text{DnaA}\right]$
(15)	$\frac{d\left[\text{GcrA}\right]}{dt}=k_{\text{s},\text{GcrA}}\cdot gcrA-k_{\text{d},\text{GcrA}}\cdot \left[\text{GcrA}\right]$
(16)	$\frac{d\left[\text{SciP}\right]}{dt}=k_{\text{s},\text{SciP}}\cdot sciP-k_{\text{d},\text{SciP}}\cdot \left[\text{SciP}\right]$
(17)	$\frac{d\left[\text{CtrA}\right]}{dt}=k_{\text{s},\text{CtrA}}\cdot ctrA-(k_{\text{d},\text{CtrA}}+\frac{k_{\text{d},\text{CtrA-ClpXP}}\cdot {\left[\text{Complex}3\right]}^{2}}{J_{\text{d},\text{CtrA-CplXP}}^{2}+{\left[\text{Complex}3\right]}^{2}})\cdot \left[\text{CtrA}\right]-k_{\text{phoCtrA}}\cdot \left[\text{CckA}\sim \text{P}\right]\cdot \left[\text{CtrA}\right]+k_{\text{dephoCtrA}}\cdot \left[\text{CtrA}\sim \text{P}\right]$
(18)	$\frac{d\left[\text{CtrA}\sim \text{P}\right]}{dt}=-(k_{\text{d},\text{CtrA}}+\frac{k_{\text{d},\text{CtrA-ClpXP}}\cdot {\left[\text{Complex}3\right]}^{2}}{J_{\text{d},\text{CtrA-ClpXP}}^{2}+{\left[\text{Complex}3\right]}^{2}})\cdot \left[\text{CtrA}\sim \text{P}\right]+k_{\text{phoCtrA}}\cdot \left[\text{CckA}\sim \text{P}\right]\cdot \left[\text{CtrA}\right]-k_{\text{dephoCtrA}}\cdot \left[\text{CtrA}\sim \text{P}\right]$
	**Equations of protease complexes**
(19)	$\frac{d\left[\text{CckA}\sim \text{P}\right]}{dt}=k_{\text{phoCckA}}\cdot (\text{CckAT}-[\text{CckA}∼\text{P]})-k_{\text{dephoCckA}}\cdot (1+\alpha_{\text{cdG}}\cdot \left[\text{cdG}\right])\cdot \left[\text{CckA}\sim \text{P}\right]$
(20)	$\frac{d\left[\text{Complex}1\right]}{dt}=k_{1}^{+}\cdot \left[\text{ClpXP}\right]\cdot \left[\text{CpdR}\right]-k_{1}^{-}\cdot \left[\text{Complex}1\right]-k_{2}^{+}\cdot \left[\text{Complex}1\right]\cdot [\text{RcdA}]+k_{2}^{-}\cdot \left[\text{Complex}2\right]$
(21)	$\frac{d\left[\text{CpdR}\right]}{dt}=k_{\text{s},\text{CpdR}}\cdot \frac{[\text{CtrA}∼{\text{P]}}^{2}}{J_{\text{a},\text{CpdR-CtrA}}^{2}+[\text{CtrA}∼{\text{P]}}^{2}}-k_{\text{d},\text{CpdR}}\cdot \left[\text{CpdR}\right]\cdot \frac{\left[\text{Complex}1\right]}{J_{\text{d},\text{CpdR}}+\left[\text{Complex}1\right]}+k_{1}^{-}\cdot \left[\text{Complex}1\right]-k_{1}^{+}\cdot \left[\text{ClpXP}\right]\cdot \left[\text{CpdR}\right]+k_{\text{dephos},\text{CpdR}}\cdot [\text{CpdR}∼\text{P]}-k_{\text{phos},\text{CpdR}}\cdot \left[\text{CckA}\sim \text{P}\right]\cdot \left[\text{CpdR}\right]$
(22)	$\frac{d[\text{CpdR}∼\text{P]}}{dt}=-k_{\text{d},\text{CpdR}}\cdot \left[\text{CpdR}\sim \text{P}\right]\cdot \frac{\left[\text{Complex}1\right]}{J_{\text{d},\text{CpdR}}+\left[\text{Complex}1\right]}+k_{\text{phos},\text{CpdR}}\cdot \left[\text{CckA}\sim \text{P}\right]\cdot \left[\text{CpdR}\right]-k_{\text{dephos},\text{CpdR}}\cdot \left[\text{CpdR}\sim \text{P}\right]$
(23)	$\frac{d\left[\text{Complex}2\right]}{dt}=k_{2}^{+}\cdot \left[\text{Complex}1\right]\cdot [\text{RcdA}]-k_{2}^{-}\cdot \left[\text{Complex}2\right]+k_{3}^{-}\cdot \left[\text{Complex}3\right]-k_{3}^{+}\cdot {\left[\text{c-di-GMP}\right]}^{2}\cdot \left[\text{Complex}2\right]$
(24)	$\frac{d\left[\text{RcdA}\right]}{dt}=k_{\text{s},\text{RcdA}}\cdot \frac{{\left[\text{CtrA}\sim \text{P}\right]}^{2}}{J_{\text{a},\text{RcdA-CtrA}}^{2}+{\left[\text{CtrA}∼\text{P}\right]}^{2}}-k_{\text{d},\text{RcdA}}\cdot \left[\text{RcdA}\right]\cdot \frac{\left[\text{Complex}1\right]}{J_{\text{d},\text{RcdA}}+\left[\text{Complex}1\right]}$
(25)	$\frac{d\left[\text{Complex}3\right]}{dt}=k_{3}^{+}\cdot {\left[\text{c-di-GMP}\right]}^{2}\cdot \left[\text{Complex}2\right]-k_{3}^{-}\cdot \left[\text{Complex}3\right]$
(26)	$\frac{d\left[\text{PleD}\right]}{dt}=k_{\text{s},\text{PleD}}\cdot \frac{{\left[\text{CtrA}\sim \text{P}\right]}^{2}}{J_{\text{a},\text{PleD-CtrA}}^{2}+{\left[\text{CtrA}\sim \text{P}\right]}^{2}}-k_{\text{d},\text{PleD}}\cdot \left[\text{PleD}\right]-k_{\text{phosPleD}}\cdot \left[\text{PleD}\right]+k_{\text{dephoPleD}}\cdot \left[\text{PleC}\right]\cdot \left[\text{PleD}\sim \text{P}\right]$
(27)	$\frac{d\left[\text{PleD}\sim \text{P}\right]}{dt}=k_{\text{phosPleD}}\cdot \left[\text{PleD}\right]-k_{\text{dephoPleD}}\cdot \left[\text{PleC}\right]\cdot \left[\text{PleD}\sim \text{P}\right]$
(28)	$\frac{d\left[\text{PdeA}\right]}{dt}=k_{\text{s},\text{PdeA}}\cdot \frac{{\left[\text{CtrA}\sim \text{P}\right]}^{2}}{J_{\text{a},\text{PdeA-CtrA}}^{2}+{\left[\text{CtrA}\sim \text{P}\right]}^{2}}-k_{\text{d},\text{PdeA}}\cdot \left[\text{PdeA}\right]\cdot \frac{\left[\text{Complex}1\right]}{J_{\text{d},\text{PdeA}}+\left[\text{Complex}1\right]}$
(29)	$\frac{d\left[\text{cdG}\right]}{dt}=k_{\text{s},\text{cdG}}\cdot (1+\alpha_{\text{PleD}}\cdot \left[\text{PleD}\right])\cdot \frac{J_{\text{i},\text{cdG-cdG}}^{2}}{J_{\text{i},\text{cdG-cdG}}^{2}+{\left[\text{cdG}\right]}^{2}}-k_{\text{d},\text{cdG}}\cdot (1+\alpha_{\text{PdeA}}\cdot \left[\text{PdeA}\right])\cdot \left[\text{cdG}\right]+k_{3}^{-}\cdot \left[\text{Complex}3\right]-k_{3}^{+}\cdot \left[\text{cdG}\right]\cdot \left[\text{Complex}2\right]$

**Fig 6:**
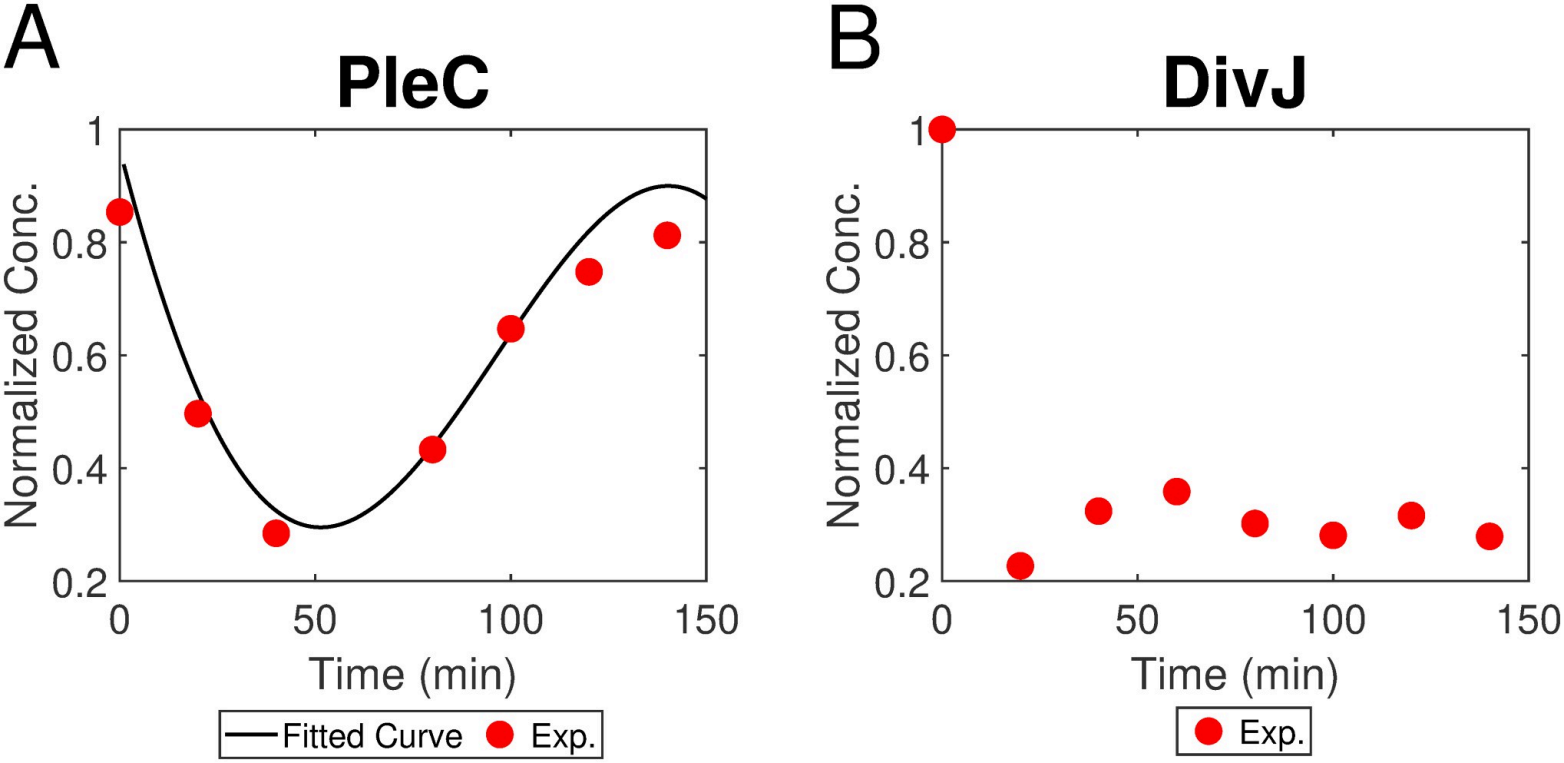
Quantification of experimental data of PleC from [35] and DivJ from [36]. (A) Black curve is fitted from experimental data by MATLAB. The fitting function is 80.09 × sin(0.013*t* + 1.74) + 78.77 × sin(0.013*t* + 4.85). (B) Experimental data of DivJ indicates DivJ levels sharply drop during the sw-to-st transition and then almost do not change.

Only phosphorylated form of PleD is active to catalyze the synthesis of cdG [32]. As the phosphorylation of PleD is controlled by more than three enzymes, including PleC, DivJ, CckN, and at least one unknown kinase [33, 34], it is complicated to thoroughly involve phosphorylation pathway of PleD. We initially assumed that phosphorylated PleD has a similar trend over cell cycle with total PleD and used total PleD as the synthetase of cdG; but cdG simulation in predivisional cell was super high, inconsistent with experiments, although both PleD and PdeA fit data well. We hypothesize PleD∼P is relatively low in predivisional cell due to the regulation of its main phosphatase PleC and kinase DivJ. To verify our hypothesis, we quantify western blots of DivJ and PleC over cell cycle using ImageJ [35, 36] (Fig 6). Experimental data indicates that DivJ almost does not change during the cell cycle and PleC is high in predivisional cell. Therefore, it is reasonable that PleD∼P decreases in predivisional cell because of high phosphatase activity of PleC. We fit PleC data points with trigonometric functions: 80.09 × sin(0.013*t* + 1.74) + 78.77 × sin(0.013*t* + 4.85) (Fig 6A). The function of PleC is then introduced into our model to represent the PleC level regulating the phosphorylation of PleD.

#### Module 3: Chromosome replication and methylation

We build the module for DNA replication following the recognized principle in Li et al’s work [4], which consists of initiation, elongation, and termination phases. During the sw-to-st transition, *C. crescentus* requires high levels of DnaA and low levels of CtrA to initiate DNA replication [13]. As DNA synthesis proceeds, the fully methylated chromosome becomes hemimethylated due to the semiconservative replication. Replication will not be initiated again until CcrM re-methylates *Cori* once more [13]. Additionally, the master regulator genes *ctrA*, *dnaA*, and *ccrM* have CcrM-targeted sequence GANTC in their promoters (see Fig 2). Therefore, the methylation state of these genes are likely influenced by CcrM abundance and the progression of replication. Taken together, the initiation of DNA replication occurs when CtrA concentration is low, DnaA concentration is high, and *Cori* is fully methylated. Once initiated, DNA replication continues in a bidirectional manner along circular chromosomes and terminates in the late predivisional cell [37]. Finally, the newly replicated chromosomes are separated into two daughter cells with the Z-ring constriction.

We use variables *Ini* and *Elong* to model the initiation and elongation phase of DNA replication, respectively, where *Elong* was built by Li et al [4] (Table 1, Eqs. 1-2). *Ini* = *P*_elong_ signals the end of initiation and the beginning of the elongation phase, where *P*_elong_ = 0.05 [13]. The initial value of *Elong* is 0.1 (2 × *P*_*elong*_) because the chromosome replication of *C. crescentus* is bidirectional. DNA replication is terminated when *Elong* = 1 and we reset *Elong* = 0 once replication is terminated. *h*, indicating the probability of hemimethylation [4], is introduced in this study to describe the methylation influences on transcript rate (see Table 1, Eqs. 7-12). As the position of *dnaA* is very close to *Cori* (see Fig 2), ‘(2 − *h*_*Cori*_)’ is used to represent the methylation effect of *dnaA* [4] and *dnaA* transcription rate reduces to half when it is hemimethylated [5]. *I* is introduced for a time delay. The chromosomes are separated with Z-ring constriction; however, the Z-ring event is not modeled in this study. Experiments indicate the S-phase period of *Caulobacter* is approximately 90 min. Here, we introduce a variable *Zring* to control the timing of Z-ring constriction and cell division. The increase rate of *Zring* is set as a particular constant to control the time for *Zring* rising from 0 to 1; and we use the time event *Zring* = 1 (Table 2) to signal the separation of chromosomes, where the count of chromosomes goes from 2 to 1 [6]. Throughout the execution of our simulation, several events representing cellular phenomena, including time points of replication initiation and chromosome segregation, can be triggered given particular conditions (summarized in Table 2).

**Table 2:** Event list.

Event description	Condition	Change(s)
DNA replication initiates	*Ini* = *P*_*elong*_	*Ini* = 0, *Elong* = 0.05, *DNA* = 1.05, *k*_*s*,*Zring*_ = 0.011, *h*_*Cori*_ = 1
replication fork passes *ccrM* locus	*Elong* = 0.2	*h*_*ccrM*_ = 1
replication fork passes *ctrA* locus	*Elong* = 0.375	*h*_*ctrA*_ = 1
DNA elongation terminates	*Elong* = 1	*Elong* = 0
Z-ring constriction	*Zring* = 1	*Zring* = 0, *k*_*s*,*Zring*_ = 0, *DNA* = 1

### Model derivation

Some proteins are not uniformly distributed in *Caulobacter* cells (see Fig 1). As we focus on temporal dynamics of regulators and their contributions to cell development, we ignore the non-uniform distributions and assume the whole cell is well-mixed at this stage. We use the law of mass action to describe the general synthesis/degradation and binding/unbinding processes, while protein effects–activation and inhibition–are described by Hill functions. To be more specific,

$$\begin{array}{c} x\overset{k_{\text{s},\text{X}}}{⟶}\text{X}\overset{k_{\text{d},\text{X}}}{⟶}\emptyset \end{array}$$ (1)

is converted as

$$\begin{array}{c} \frac{d[\text{X}]}{dt}=k_{\text{s},\text{X}}\cdot x-k_{\text{d},\text{X}}\cdot [\text{X}] \end{array}$$ (1)

where X represents protein, *x* is the mRNA of X, *k*_s,X_ is the rate constant of synthesis, and *k*_d,X_ indicates the rate constant of degradation.

$\text{A}+\text{B}\overset{{\text{k}}^{+}\left(\text{bind}\right)}{\underset{{\text{k}}^{-}\left(\text{unbind}\right)}{⇌}}\text{C}$ is converted as

$$\begin{array}{c} \frac{d[\text{C}]}{dt}=k^{+}\cdot [\text{A}]\cdot [\text{B}]-k^{-}\cdot [\text{C}] \end{array}$$ (2)

A binds to B to produce C, where *k*^+^ and *k*^−^ represent binding and unbinding rates, respectively. Activation and inhibition effects are described by Hill functions as follow:

$$\begin{array}{c} H_{\text{a}}\left(\text{X}\right)=\frac{{\text{X}}^{n}}{J_{a,x}^{n}+{\text{X}}^{n}},\;H_{\text{i}}\left(\text{X}\right)=\frac{J_{\text{i},x}^{n}}{J_{\text{i},x}^{n}+{\text{X}}^{n}}, \end{array}$$ (3)

where *H*_a_(X) indicates activation, and *H*_i_(X) indicates inhibition. Variables *n*, *J* represent the corresponding Hill coefficient and the microscopic dissociation constant, respectively.

### Model parameters

#### Experimental data

To compare our simulations with experimental observations from different publications, we first normalize experimental data to [0, 1] as follows:

$$\begin{array}{c} z_{i}=\frac{x_{i}-\text{min}\left(x_{i}\right)}{\text{max}\left(x_{i}\right)-\text{min}\left(x_{i}\right)}, \end{array}$$ (4)

where *x*_*i*_ indicates the original data point; *z*_*i*_ is the scaled normalized value of experiments. Second, considering the relative abundance of different species in experiments [38], we set different targeted ranges for different species in the model. For example, the abundances of DnaA and CcrM are relatively low while those of CtrA and SciP are relatively high in experiments [38] and our simulations. For the figures in the Result section, the normalized experimental data are scaled to the range of our simulations to evaluate the temporal dynamics.

#### Parameter description

All 86 parameters used in this study are summarized in Table 3. Among them, seven parameters are obtained from previous experimental or modeling publications (see parameters marked with * in Table 3).

**Table 3:** Parameter values. Parameters marked with * are obtained from publications.

**Parameters of DNA**
Rate constants, units = min^-1^
*k*_s,Ini_ = 3.104e-4, $k_{\text{elong}}^{*}=6.53\text{e-}3$ [4], *P*_elong*_ = 0.05 [4],
*k*_m,*Cori*_ = 1.5637, *k*_m,*ccrM*_ = 2.2763, *k*_m,*ctrA*_ = 1.4645
Binding constants (dimensionless)
*J*_a,Ini_ = 1, *J*_i,Ini_ = 1.4565, *J*_m,*Cori*_ = 0.95, *J*_m,*ccrM*_ = 0.95, *J*_m,*ctrA*_ = 0.95
Scaling variables (dimensionless)
Θ_CtrA_ = 6.0, Θ_DnaA_ = 0.5, Θ_Cori_ = 0.308
**Parameters of mRNAs**
Rate constants, units = min^−1^
$k_{\text{s},I_{ccrM}}=0.1105$, $k_{\text{d},I_{ccrM}}=0.0696$, *k*_s,*ccrM*_ = 0.2557, *k*_d,*ccrM*_ = 0.1005,
*k*_s,*dnaA*_ = 0.199, *k*_d,*dnaA*_ = 0.0693, *k*_s,*gcrA*_ = 5.4235, *k*_d,*gcrA*_ = 0.7342,
*k*_s1,*ctrA*_ = 1.0035, *k*_s2,*ctrA*_ = 0.0937, *k*_d,*ctrA*_ = 0.0983, *k*_s,*sciP*_ = 0.583, *k*_d,*sciP*_ = 0.0523,
Binding constants (dimensionless)
*J*_a,*ccrM*-CtrA_ = 5, *J*_i,*ccrM*-SciP_ = 6, *J*_i,*dnaA*-GcrA_ = 3, *J*_a,*gcrA*-DnaA_ = 1.25, *J*_i,*gcrA*-CtrA_ = 5,
*J*_a,*ctrA*-CtrA_ = 5, *J*_a,*ctrA*-GcrA_ = 3, *J*_i,*ctrA*-CtrA_ = 8, *J*_i,*ctrA*-SciP_ = 8, *J*_a,*sciP*-CtrA_ = 5
**Parameters of master regulators**
Rate constants, units = min^−1^
*k*_s,DnaA_ = 0.0787, $k_{\text{d},\text{DnaA}}^{*}=0.07$ [54], *k*_s,GcrA_ = 0.032, $k_{\text{d},\text{GcrA}}^{*}=0.022$ [4],
*k*_s,CcrM_ = 0.0834, $k_{\text{d},\text{CcrM}}^{*}=0.07$ [55], *k*_s,SciP_ = 0.1294, *k*_d,SciP_ = 0.0673,
*k*_s,CtrA_ = 0.0404, $k_{\text{d},\text{CtrA}}^{*}=0.002$ [4], *k*_d,CtrA-ClpXP_ = 0.053
Phosphorylation constant, units = min^−1^
*k*_phos,CtrA_ = 4.2919, *k*_dephos,CtrA_ = 0.113, *k*_phos,CckA_ = 1.027, *k*_dephos,CckA_ = 0.9242
Binding constants (dimensionless)
*J*_d,CtrA-ClpXP_ = 4
**Parameters of protease complexes**
Rate constants, units = min^−1^
$k_{1}^{+}=0.6072$, $k_{2}^{+}=1.4375$, $k_{3}^{+}=170.4913$, $k_{1}^{-}=3.3013$, $k_{2}^{-}=0.8164$, $k_{3}^{-}=2.3133$,
*k*_phos,PleD_ = 0.046, *k*_dephos,PleD_ = 0.0414, *k*_s,CpdR_ = 1.2227, *k*_d,CpdR_ = 1.6152,
*k*_pho,CpdR_ = 1.1239, *k*_depho,CpdR_ = 1.3854, *k*_s,RcdA_ = 0.1642, *k*_d,RcdA_ = 0.2323,
*k*_s,cdG_ = 0.0099, *k*_d,cdG_ = 0.9893,
*k*_s,PleD_ = 0.0956, *k*_d,PleD_ = 0.1314, *k*_s,PdeA_ = 0.012, *k*_d,PdeA_ = 0.5161
Binding constants (dimensionless)
*J*_a,CpdR-CtrA_ = 15, *J*_d,CpdR_ = 6, *J*_a,RcdA-CtrA_ = 15, *J*_d,RcdA_ = 2
*J*_a,PdeA-CtrA_ = 5, *J*_d,PdeA_ = 5, *J*_a,PleD-CtrA_ = 2.5, $J_{\text{i},\text{cdG-cdG}}^{*}=0.2$ [51]
Constants (dimensionless)
CckAT = 0.3, [ClpXP] = 1, *α*_PdeA_ = 7, *α*_PleD_ = 1500, *α*_*cdG*_ = 10

The rest of the parameters are split into two groups: 1) 47 parameters (summarized in Table 4) that characterize major functionality of mRNAs and proteins, such as synthesis and degradation, are chosen for optimization; 2) the remaining 32 parameters are set with fixed values, including most dissociation constants.

**Table 4:** Parameter optimization with lower and upper bounds and starting point.

Parameter	[L,U]	Starting	Parameter	[L,U]	Starting
*k*_m,Cori_	[0.35, 5.6]	1.4	*k*_m,ccrM_	[0.35, 5.6]	1.4
*k*_m,ctrA_	[0.35, 5.6]	1.4	$k_{\text{s},I_{ccrM}}$	[0.025 0.4]	0.1
$k_{\text{d},I_{ccrM}}$	[0.016675, 0.2668]	0.0667	*k*_s,ccrM_	[0.064, 1.024]	0.256
*k*_d,ccrM_	[0.02, 0.32]	0.08	*k*_s,dnaA_	[0.0605,0.968]	0.242
*k*_d,dnaA_	[0.015, 0.24]	0.06	*k*_s,gcrA_	[1.4, 22.4]	5.6
*k*_d,gcrA_	[0.15, 2.4]	0.6	*k*_s,sciP_	[0.125, 2]	0.5
*k*_d,sciP_	[0.01, 0.16]	0.04	*k*_s1,ctrA_	[0.2475, 3.96]	0.99
*k*_s2,ctrA_	[0.0225, 0.36]	0.09	*k*_d,ctrA_	[0.02075, 0.332]	0.083
*k*_s,DnaA_	[0.01625, 0.26]	0.065	*k*_s,GcrA_	[0.007, 0.112]	0.028
*k*_s,CcrM_	[0.02125, 0.34]	0.085	*k*_s,SciP_	[0.0295, 0.472]	0.1183
*k*_d,SciP_	[0.015, 0.24]	0.06	*k*_s,CtrA_	[0.0108, 0.1728]	0.0432
*k*_d,CtrA-ClpXP_	[0.015, 0.24]	0.06	$k_{1}^{+}$	[0.15, 2.4]	0.6
$k_{1}^{-}$	[0.75, 12]	3	*k*_s,CpdR_	[0.175, 2.8]	0.7
*k*_d,CpdR_	[0.375, 6]	1.5	*k*_dephos,CpdR_	[0.25, 4]	1
*k*_phos,CpdR_	[0.25, 4]	1	$k_{2}^{+}$	[0.275, 4.4]	1.1
$k_{2}^{-}$	[0.25, 4]	1	*k*_s,RcdA_	[0.0375, 0.6]	0.15
*k*_d,RcdA_	[0.05, 0.8]	0.2	$k_{3}^{+}$	[35, 560]	140
$k_{3}^{-}$	[0.5, 8]	2	*k*_s,cdG_	[0.0025, 0.04]	0.01
*k*_d,cdG_	[0.25, 4]	1	*k*_s,PleD_	[0.025, 0.4]	0.1
*k*_d,PleD_	[0.0375, 0.6]	0.15	*k*_phosPleD_	[0.01, 0.16]	0.04
*k*_dephosPleD_	[0.01, 0.16]	0.04	*k*_s,PdeA_	[0.0025, 0.04]	0.01
*k*_d,PdeA_	[0.125, 2]	0.5	*k*_phoCtrA_	[1.25, 20]	5
*k*_dephoCckA_	[0.25, 4]	1	*k*_dephoCtrA_	[0.025, 0.4]	0.1
*k*_phoCckA_	[0.25, 4]	1			

#### Multiobjective optimization

Let $\chi \in R^{p}$, *p* = 47 be the vector of parameters to be estimated in the *caulobactor* cell cycle model. For this optimization problem, we focus on two aspects: the quantitative difference between experimental data and simulated results, and the cell cycle time, both for wild type cells. The reasoning behind the two objectives is that the experimental data have inconsistent concentration levels between the beginning (*t* = 0 min) and ending (*t* = 150 min) states of the cell cycle, whereas our model must be consistent to ensure stable cell cycle regulation. This is also validated in our initial optimization test using a single objective function, where we observe minimizing the difference in species concentration results in high deviation in cell cycle time, and vice versa. Due to this conflict, we cannot use the common scalarization scheme to sum up the two objectives using weights, i.e., *F*(*χ*) = *w*_1_*f*_1_(*χ*) + *w*_2_*f*_2_(*χ*). Our parameter optimization is therefore defined as a multiobjective optimization problem (MOP). The two objective functions are:

$$\begin{array}{c} f_{1}\left(\chi \right)=\frac{1}{nm}\sum_{i}^{n}\sum_{j}^{m}{\left(x_{i,j}-y_{i,j}\right)}^{2},\;f_{2}\left(\chi \right)=\left|T_{c}-150\right|, \end{array}$$ (5)

where *x*_*i*,*j*_ denotes the simulated concentration of species *i* at time *j*, *y*_*i*,*j*_ denotes the experimental data of species *i* at time *j*, and *T*_*c*_ is the simulated cell cycle time. Here, we have the experimental data for *n* = 15 species (see Table 5) and the number of data points *m* varies for different species. Note that we only use available observations of *C. crescentus* wild type (WT) cells for parameter fitting. The mutant cases of *C*. cell are used as our model validation. The optimization problem to be solved is

$$\begin{array}{c} \underset{\left[L,U\right]}{\text{min}}f_{1}\left(\chi \right),\;\underset{\left[L,U\right]}{\text{min}}f_{2}\left(\chi \right), \end{array}$$ (6)

where [*L*, *U*] is a search box in $R^{p}$ for model parameters. See the lower and upper bounds of parameters in Table 4.

**Table 5:** Sources for experimental data used to evaluate our models.

Species	Data source	Species	Data source
*ccrM*	[12]	CcrM	[47, 48]
*dnaA*	[12]	DnaA	[7, 47]
*gcrA*	[12]	GcrA	[2, 7]
*sciP*	[12]	SciP	[2]
*ctrA*	[12]	CtrA	[47, 48]
CpdR	[25]	RcdA	[44]
PleD	[45]	PdeA	[45]
cdG	[31]		

**Fig 7:**
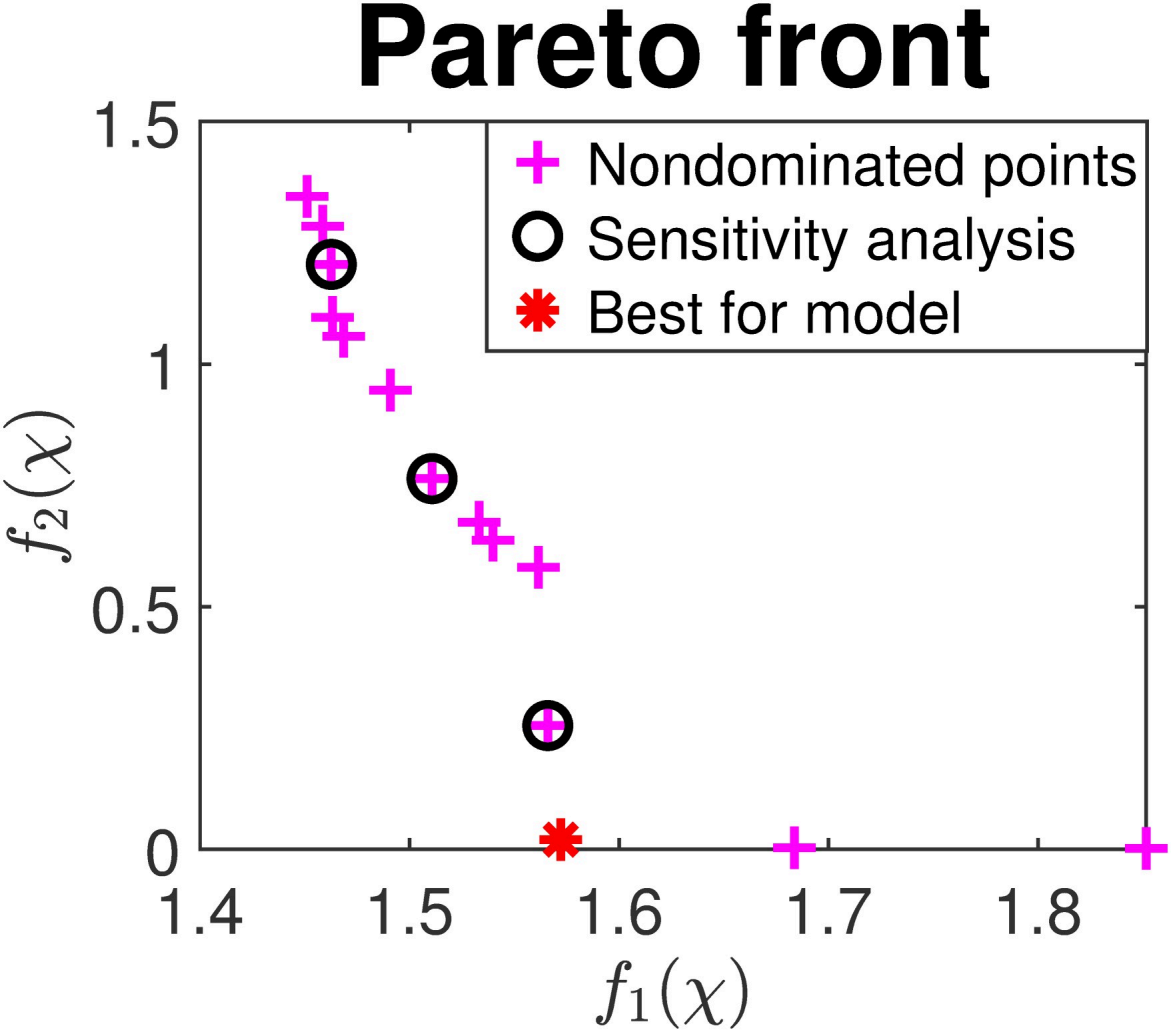
Pareto front returned by NSGA-II and VTMOP. *f*_1_(*χ*) and *f*_1_(*χ*) are the two objective function values. The red point is selected as the best parameter set for our model.

We apply two MOP algorithms to our optimization problem for comparison: one is the widely used nondominated sorting genetic algorithm (NSGA-II) [39]; the other is the more recent VTMOP [40] based on VTdirect [41] and QNSTOP [42, 43] that uses response surface and trust region methodologies, and an adaptive weighting scheme. Initial values in Table 6 are the levels of corresponding variables used as the beginning state of each simulated cell cycle. Fig 7 shows the combined Pareto front from both methods after multiple runs with different optimization settings. Observe that the Pareto front is a nonconvex curve, showing that the multiobjective optimization problem is very difficult. The best parameter estimates are found by VTMOP and listed in Table 3, with *f*_1_ = 1.57 and *f*_2_ = 0.02. The sensitivity of parameters is 18% for experimental data fitting (*f*_1_) and 72% for cell cycle time (*f*_2_) if we perturb the parameters of three Pareto points (marked as black circle in Fig 7) by 10%. Note that the sensitivity of the second objective is large when *f*_2_ is very close to zero, thus points near zero are not included in the analysis. The root mean square error (goodness of fit) is RMS(*f*_1_) ≈ 1.51, RMS(*f*_2_) ≈ 0.84 for the three selected Pareto points.

**Table 6:** Initial values of model variables.

**DNA variables**	**Initial values**	**Master regulator vars**.	**Initial values**
*Ini*	0.0383	CcrM	0.435
*Elong*	0	DnaA	2.638
*DNA*	1	GcrA	3.841
*Count*	1	SciP	12.485
*Zring*	0	CtrA	1.973
*h_Cori_*	0	CtrA∼P	3.960
*h_ccrM_*	0		
*h_ctrA_*	0		
**mRNA variables**	**Initial values**	**Protease complex vars**.	**Initial values**
*ccrM*	0.173	Complex 1	0.211
*dnaA*	3.154	CpdR	1.045
*gcrA*	4.525	CpdR∼P	0.042
*sciP*	6.335	Complex 2	0.187
*ctrA*	0.658	RcdA	0.789
		Complex 3	3.407
		CckAP	0.042
		cdG	0.511
		PleD	0.526
		PleD∼P	0.663
		PdeA	0.228

## Results

Integrating the hierarchical proteolysis (Fig 5) into the master regulatory network (Fig 3), we propose a model to capture the temporal dynamics of cell cycle regulators and glean insights about bacterial protein proteolysis systems. Non-uniform distributions of molecules in space are ignored at this stage.

### Our model accurately describes gene transcription patterns and temporal dynamics of key regulators during the replication cycle of *Caulobacter* wild type cells

#### Chromosome replication and methylation

**Fig 8:**
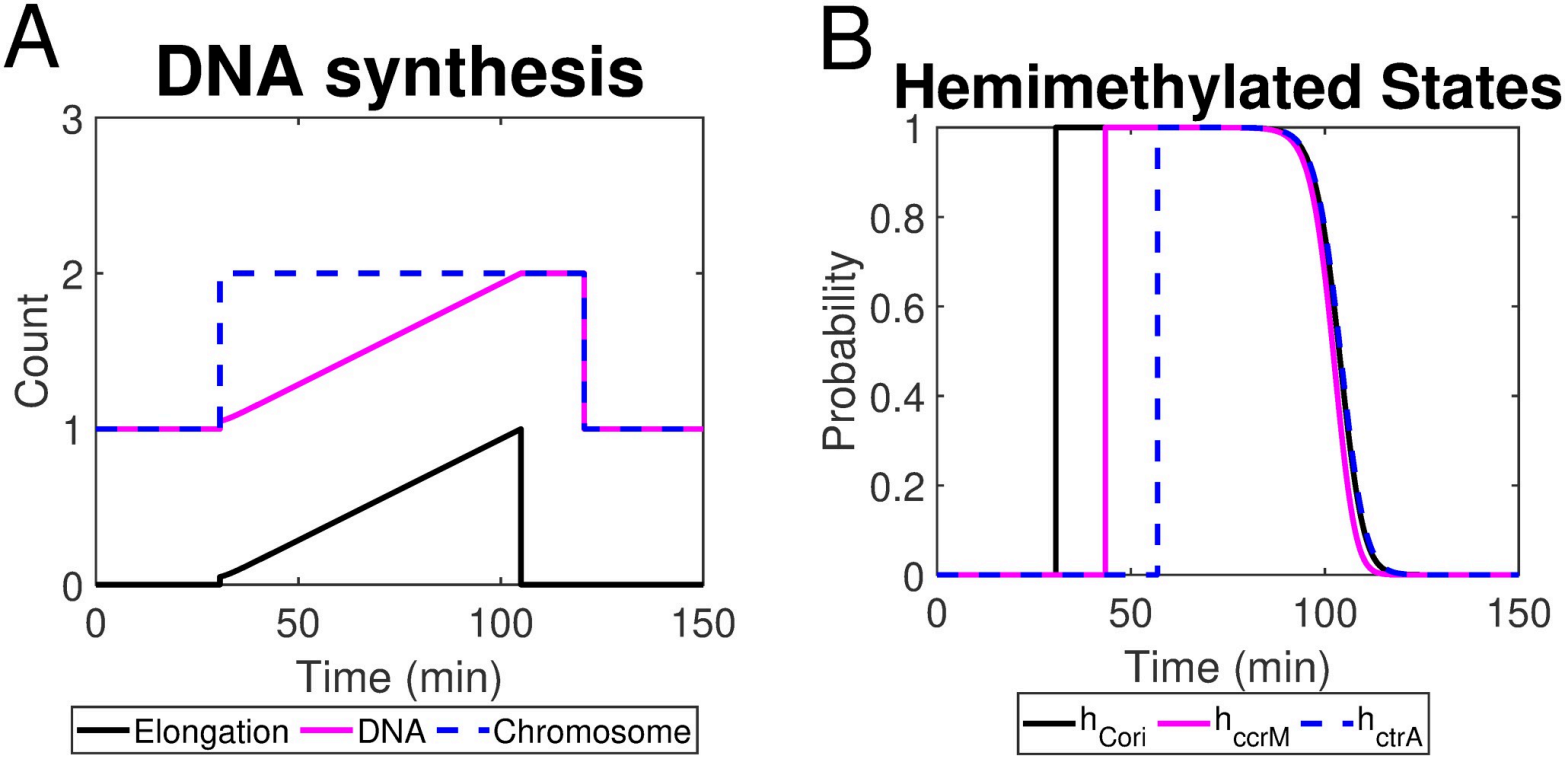
(A) Simulated chromosome/DNA replication and elongation process; (B) The probability of loci (*Cori*, *ccrM*, *ctrA*) being hemimethylated in a single cell cycle.

We follow the DNA replication process as the rationale to formulate a set of ordinary differential equations (ODEs) modeling initiation, elongation, and termination of DNA replication as well as methylation states, as shown in Table 1 (Eqs. 1-6). The initiation of DNA replication requires a fully methylated state (both strands methylated, h_*_ = 0), while semiconservative replication creates two hemimethylated copies of genes. As such, the variables h_*_ in our model spike when the corresponding gene is being replicated (Fig 8). Later in the cell cycle, the hemimethylated copies (h_*_ = 1) are re-methylated by CcrM, returning to the fully methylated state. Therefore, h_*_ then plunge as the newly created, hemimethylated copies become fully methylated by CcrM. The CcrM-dependent methylation in the control system ensures DNA replication initiates once per cell cycle.

#### The proteolysis of CtrA is controlled by hierarchical protease complexes

**Fig 9:**
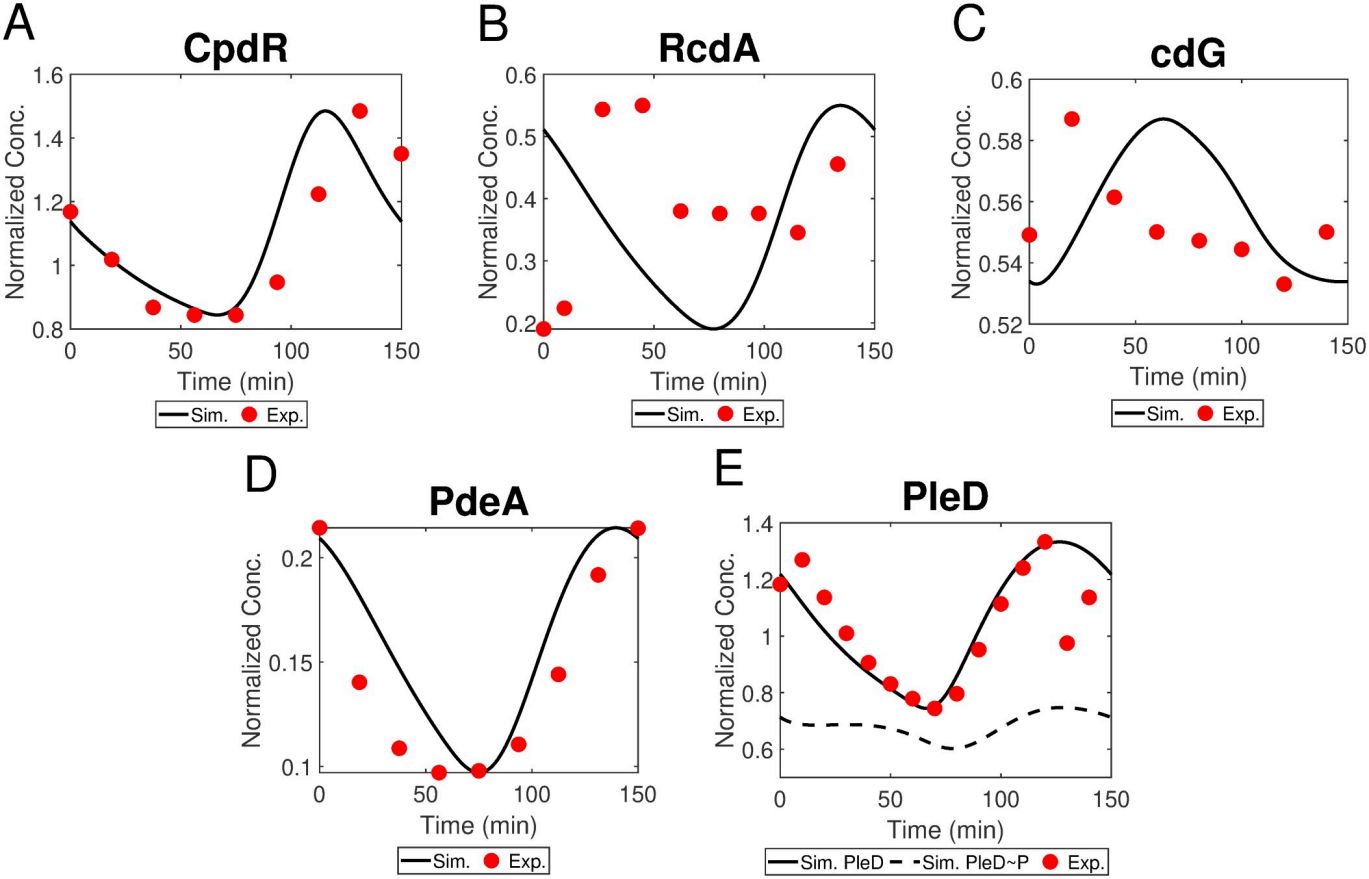
(A-E) The dynamics of total CpdR, RcdA, cdG, PdeA, total PleD, and PleD∼P in simulation with the corresponding experimental data. Experimental data of CpdR is from Iniesta et al. [25], RcdA is from McGrath et al. [44], cdG is from Abel et al. [31], and PdeA as well as total PleD are from Abel et al. [45].

In addition to replication and transcription, we investigate the proteolysis regulation of CtrA, the essential component of cell cycle control system, and explore the contribution of the conserved proteolysis module. Based on the hierarchical diagram of protease complexes (Fig 5), we use ODEs to simulate the temporal dynamics of three classes of protease complexes (Eqs. 20-29 of Table 1). Since there is no experimental data of protease complexes, we evaluate our simulations using western blots of CpdR, RcdA, PleD, PdeA, and cdG [14, 44, 45] (see Table 5), where numerical values are extracted by ImageJ or GetData, shown as the red circles in Fig 9. Those proteins are essential components of ClpXP-dependent proteolysis system.

Our simulated CpdR, PleD and PdeA match well the experimental dynamics (see Fig 9). The general trend of modeled RcdA and cdG agrees with experiments, whereas cdG peaks a little bit late compared with experimental data. The discrepancy may derive from other regulatory enzymes of cdG or PleD which are not involved in our current model. As most proteins involved in protease complexes are modeled reasonably, We use the hierarchical model to simulate the cyclic proteolysis of CtrA (Eq. 17 of Table 1). In addition to degradation regulation, the hierarchical model influences the phosphorylation of CtrA via cdG and CckA, while phosphorylated CtrA in turn impacts the expression of components involved in degradation module, including *cpdR*, *rcdA*, and *pleD*.

#### Temporal dynamics of mRNA and master regulators

**Fig 10:**
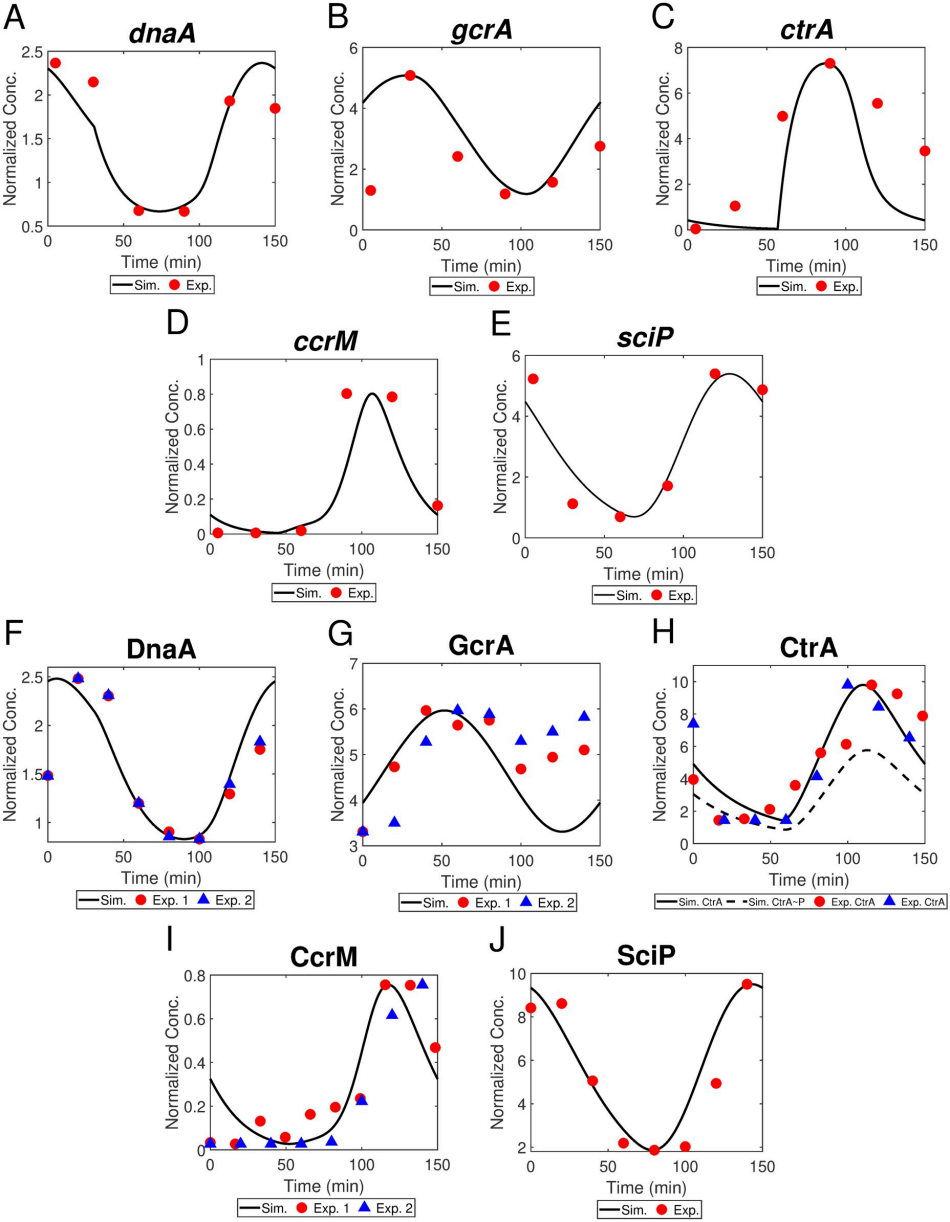
(A-E) Experimental mRNA concentration of *dnaA*, *gcrA*, *ctrA*, *ccrM*, *sciP* (curves) with corresponding simulated data (red circles, from Schrader et al. [12]), and (F-J) simulated protein concentration of DnaA, GcrA, total CtrA (CtrA∼P), CcrM, SciP (curves) with experimental data (circles or triangles) over a single cell cycle. For the sources of experimental data, DnaA data is from Shen et al. [47] and Collier et al. [7]; GcrA data is from Collier et al. [7] and Tan et al. [2]; CtrA data and CcrM data are both from Reisenauer et al. [48] and Shen et al. [47]; and SciP data is from Tan et al. [2].

**Fig 11:**
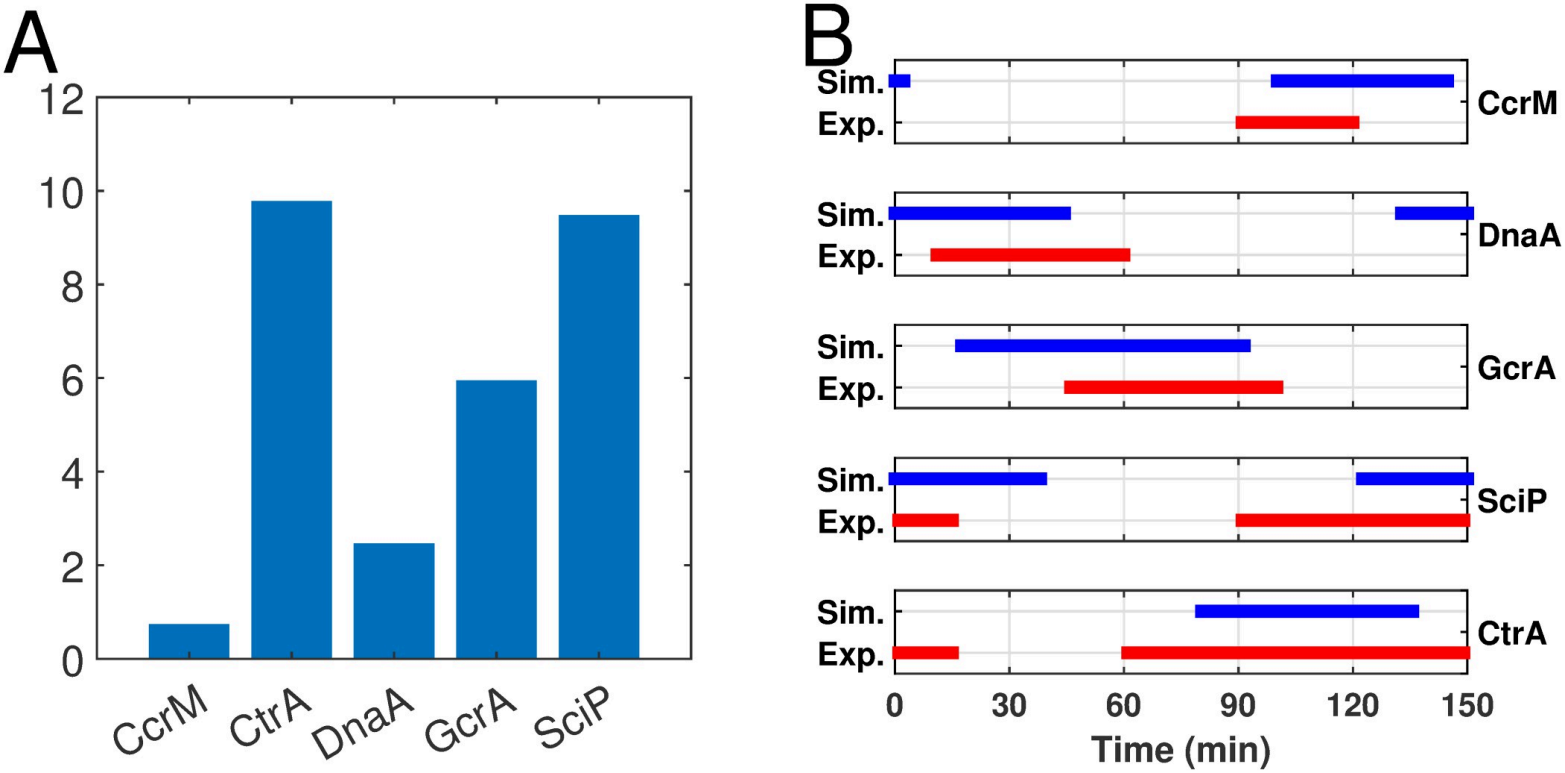
(A) Relative maximum concentrations of master regulators across one swarmer cell cycle. (B) Abundance of five master regulators (CcrM, CtrA, DnaA, GcrA, and SciP) from simulated results and experimental data. Horizontal bars represent the time periods of protein abundance across the swarmer cell cycle. Blue bars indicate the time frame where simulated protein levels are above the mid-range concentrations and red bars are the corresponding experimental data from [24].

We convert the regulatory network diagram in Fig 3 into ODEs shown in Table 1 (Eqs. 7-18) to simulate the temporal dynamics of five master regulators and their mRNA. The proposed hierarchical protease complexes are applied to simulate the cyclic degradation of CtrA. Fig 10A–10E and 10F–10J exhibit the comparisons between simulations (black curves) in our model and experimental data (red circles and blue triangles) for mRNA (*dnaA*, *gcrA*, *ctrA*, *ccrM*, and *sciP*) and protein (DnaA, GcrA, CtrA, CcrM, and SciP) levels, respectively. In general, our simulations fit the experimental observations well. As we capture the genetic information flowing from mRNA to proteins, the protein concentration curves generally resemble the corresponding mRNA abundance curves. *dnaA* transcription is reduced by hemi-methylation state, which in part explains the dip in our simulation of *dnaA* during DNA replication (*t* ∈ [30, 110] min in Fig 10A). Additionally, the expression of *dnaA* is activated by CtrA [5] and inhibited by GcrA [46]. Thus, the high levels of GcrA and low levels of CtrA during sw-to-stalk transition reinforce the decrease of *dnaA* expression (Fig 10A, 10G and 10H). When the replication fork passes *ccrM* and *ctrA* right before and after 50 min, *h*_*ccrM*_ and *h*_*ctrA*_ are switched from 0 to 1 (Fig 8), which explains the increase of CcrM (*ccrM*) and CtrA (*ctrA*) at the corresponding time (Fig 10B, 10D, 10I and 10H). Meanwhile, the high levels of activator GcrA and low levels of inhibitor SciP amplify the increase of *ctrA*. DnaA and CtrA collaborate to regulate the initiation of DNA replication: 1) during sw-to-st transition, initiator DnaA is high and suppressor CtrA is low, allowing the cell to initiate replication; 2) during DNA replication, DnaA keeps low and CtrA is high, avoiding another initiation of replication in the same cycle. Under the combined functions of DnaA and CtrA, the transcription of of *gcrA* increases in the beginning and decreases in the predivisional stage, which agrees with the observation of *gcrA* transcription (see Fig 10B). *sciP* expression is activated by CtrA, which is observed in our simulation as well (Fig 10E). Fig 11A shows the maximum levels of our simulated master regulators, in which the relative scales agree with experiments [38]. We summarize the simulated and observed abundance of five master regulators in a single cell cycle in a bar chart (Fig 11B), where our simulation shows similar translation patterns with experiments. Even though the experimental data comes from a variety of sources and experimental techniques, visual inspection suggests fair agreement between the timing of master regulator abundance in simulation and experimental data.

One objection worth noting is that some of our simulations deviate from the experimental data at the beginning or the end of the cell cycle. For example in Fig 10C, at the end of the cell cycle, the expression level of *ctrA* is considerably lower in our simulation than the experimental data suggest. Additionally, this type of discrepancy can be witnessed in the simulated GcrA, where the simulated level is lower than experimental observations after *t* = 100 min (Fig 10G). This disagreement stems from a limitation that the simulated endpoint has to be equal to the starting point (*t* = 0 min), because we do not model the asymmetrical heritage of two distinct daughter cells after progenies are completely separated. Although there are several mismatches between the simulation and experiments, our model fits most data points and captures key trends during cell cycle, such as the dynamics of regulators during sw-to-st transition. Our model exhibits that key regulators interact with each other through transcription, degradation, and phosphorylation regulations to determine the timing of cell differentiation and reproduction.

### Hierarchical protease complexes contribute to the timed cell cycle progression

**Fig 12:**
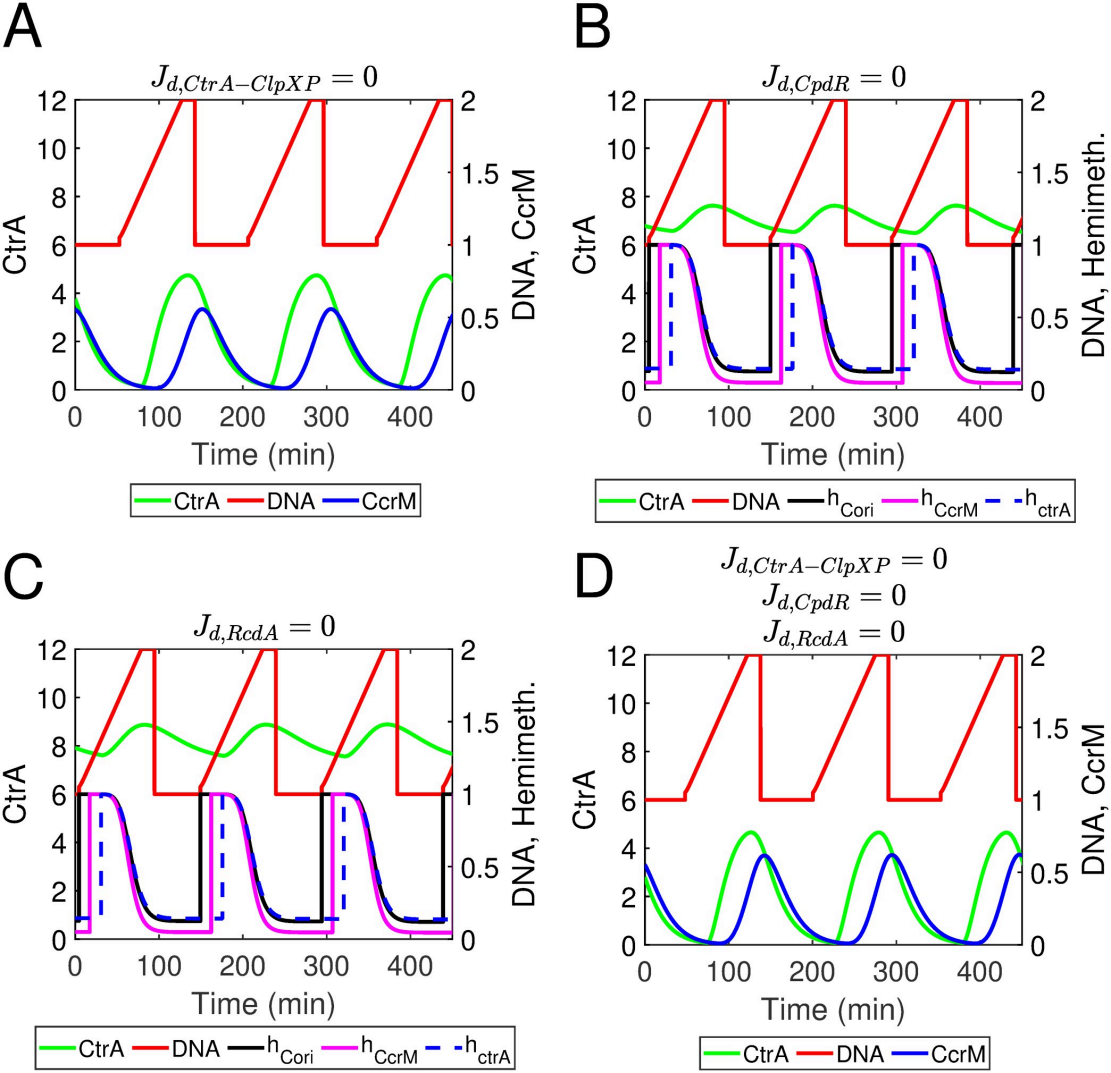
(A-D) Simulated results of mutating the cyclic proteolysis of CtrA, CpdR, or/and RcdA. (A). *J*_d,CtrA−ClpXP_ = 0 indicates the cyclic proteolysis of CtrA is replaced by a constant. (B). *J*_d,CpdR_ = 0 indicates the cyclic proteolysis of CpdR is replaced by a constant. (C). *J*_d,RcdA_ = 0 indicates the cyclic proteolysis of RcdA is replaced by a constant. (D). The cyclic proteolysis of CtrA, CpdR, and RcdA are all mutated as constant degradation.

Our modeled hierarchical cyclic proteolysis module performs well in the simulation of CtrA. Here, we explore the contribution of this module for cell development. We replace the cyclic proteolysis by a constant for CtrA, CpdR, and RcdA, separately, setting *J*_d,CtrA−ClpXP_, *J*_d,CpdR_, or *J*_d,RcdA_ as 0. In the simulation of *J*_d,CtrA−ClpXP_ = 0, where the degradation rate of CtrA is constant, the system still oscillates during cell cycles whereas the amplitude of CtrA and SciP shows noteworthy reduces. The cycle time increases, resulting in delays of master regulators in simulation, including CtrA and CcrM (Fig 12A). With a constant degradation of CpdR or RcdA, simulations show severe defects, especially for the dynamics of CtrA. The oscillation of CtrA almost disappears and methylation states are abnormal under these conditions (Fig 12B and 12C). In summary, the cyclic proteolysis deriving from the hierarchical protease complexes shows significant impacts on the system. We further replace all cyclic complexes with constants, setting *J*_d,CtrA−ClpXP_, *J*_d,CpdR_, and *J*_d,RcdA_ as 0 simultaneously. The corresponding simulation is similar with the *J*_d,CtrA−ClpXP_ = 0 mutant, which shows delayed cell cycles and reduced amplitudes of several species (Fig 12D). Simulations of these cyclic proteolysis mutants suggest the cyclic proteolysis of CtrA is key to regulate the entire system, because both deletion (*J*_d,CtrA−ClpXP_ = 0) and changes (*J*_d,CpdR_ = 0 and *J*_d,RcdA_ = 0) of CtrA cyclic degradation would screw up the dynamics pattern of both master regulators and their mRNA. Moreover, the system is more sensitive without the cyclic proteolysis module. We increased the degradation rate of CtrA to 5-fold for system with and without the cyclic proteolysis module; wild type system still has an acceptable cell cycle while cyclic proteolysis mutant systems have severe deficiencies. Taken together, our model suggests the hierarchical cyclic proteolysis module contributes the timed cell cycle and robustness of the *Caulobacter* system.

### Our model captures major phenotypes of mutant strains

**Fig 13:**
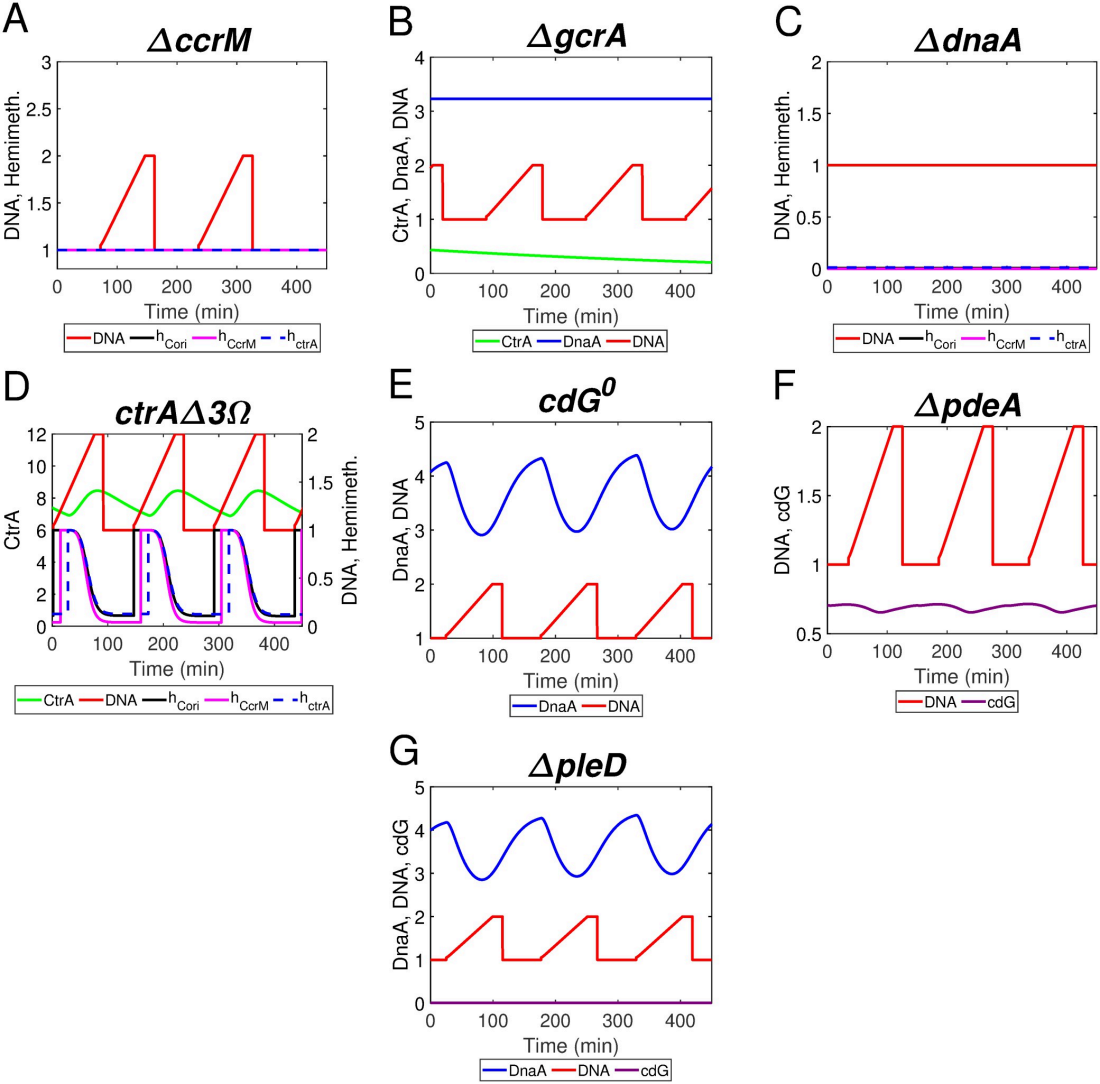
(A-G) Simulated results of mutant strains:Δ*ccrM*, Δ*gcrA*, Δ*dnaA*, *ctrA*Δ3Ω, cdG^0^, Δ*pdeA*, and Δ*pleD*. In knock out mutant simulations, we set *k*_s,i_ = 0, where i indicates corresponding species including *ccrM*, *gcrA*, *dnaA*, cdG, PdeA, and PleD. In the simulation of *ctrA*Δ3Ω, the cyclic proteolysis rate of CtrA is reduced to 10%.

To further test the validity of our model, we use the same equations and initial values to simulate seven different mutant strains (Fig 13). Among these mutant strains, cell cycle of Δ*dnaA*, where *dnaA* is knocked out (*k*_s,*dnaA*_ = 0), is arrested. The other six mutant strains are all viable. Our mutant simulations correctly capture the viability of these seven mutant strains. To be more specific:

Δ*ccrM*: *ccrM* is verified to be dispensable for cell viability [10]. The doubling time is about 162±9 min, longer than that for WT. Our simulated Δ*ccrM* (*k*_s,*ccrM*_ = 0) has a 164 min cycle time, which fits the experimental observation well (Fig 13A). In our simulation, all *h* can not be returned to 0 because there is no CcrM re-methylating the chromosome. Additionally, experiments have suggested the cell cycle is also regulated by CcrM independent with GANTC motif. This study does not include the GANTC motif independent influence of CcrM, so the simulation of Δ*ccrM* only shows the potential of deleting methylation of GANTC motif by CcrM.
Δ*gcrA*: In *gcrA* knocked out strain, the doubling time is 40% longer than for the WT [10]. Our simulated *gcrA* mutant (*k*_s,*gcrA*_ = 0) has approximately 10 min longer cell cycle compared with the WT simulation (Fig 13B), which less than then the experimental observation. The gap is likely derived from the forced modeling of Z-ring constriction process, which is not explicitly modeled in this study.
*ctrA*ΔΩ3: *ctrA*ΔΩ3 contains modifications to the C-terminal amino acids of *ctrA*, resulting in a non-proteolizable CtrA allele [49]. Here, we decreases *k*_d,CtrA-ClpXP_ to 10% of WT in simulation. In Fig 13D, The average CtrA levels increase in simulation with less fluctuation because of the non-proteolizable CtrA allele. Our simulation suggests the proteolysis of CtrA is important for its cell cycle-dependent regulation. *h* in simulated *ctrA*ΔΩ3 can not decrease to 0, suggesting the levels of CcrM in *ctrA*ΔΩ3 are not sufficient to completely re-methylate chromosome, while the lower levels of CcrM is caused by higher levels of the inhibitor CtrA.

#### cdG related mutant strains

cdG^0^ mutant strain has been verified to be viable, although it shows morphology defects [32]. In Fig 13E, our simulation of cdG^0^ strain (*k*_s,cdG_ = 0) is viable and shows a horizontal shift which may result in morphology defects. The CtrA levels increase with less fluctuation which is caused by the deletion of cdG. *pleD* knocked out mutant (*k*_s,PleD_ = 0) results in a lower cdG levels (Fig 13G), which shows a similar phenotype with the simulation of cdG^0^. *pdeA* mutant increases cdG levels in simulation (Fig 13F, *k*_s,PdeA_ = 0). Both Δ*pleD* and Δ*pdeA* are viable in simulation, consistent with observations [32, 45]. Oscillations exist but shifts little bit in the simulations of these three cdG regulated mutants, as shown in Fig 13E–13G.

## Discussion

The five major regulators–DnaA, GcrA, CcrM, CtrA, and SciP–work in tandem to drive the cell cycle progression of *C. crescentus*. Here, we investigated the interactions among master regulators to study the underlying mechanisms of DNA replication, methylation, transcription, and proteolysis of cyclic regulators. We applied the central dogma of molecular biology to simulate the temporal dynamics of mRNAs and proteins. Furthermore, we mathematically built a hierarchical model to simulate protease complexes and apply this model to CtrA degradation. Two MOP approaches (NSGA-II and VTMOP) have been applied to estimate parameters in this complicated system.

In *C. crescentus*, the protease ClpXP primed by one assistant adaptor recruits additional adaptors in sequence [27]. The hierarchical adaptor assembly determines the time and location of the proteolysis of hierarchical substrates. Our hierarchical model correctly captures the key dynamics of CpdR, PleD, and PdeA; it shows fair agreement with the trend of RcdA and cdG. Additionally, the protease model performs well in modeling the proteolysis of CtrA. Deleting the hierarchical protease module causes defects of cell cycle development and protein oscillations. Considering the fast formation of the protease Complex 3 (ClpXP bound with CpdR, RcdA, PopA, and cdG), we test quasi-steady-state assumption (QSSA) for Complex 3. QSSA shows similar simulated results in both wild type cells and mutant cases, suggesting QSSA might be a good approach in reducing model complexity of biological systems. As a wide range of proteins is degraded by ClpXP protease complex, our model provides a good quantitative tool to analyze the proteolysis of these proteins in *C. crescentus*, such as TacA and ShkA. As most components of the hierarchic protease complexes are conserved in bacterial species, our model has the potential for a wide range of applications. Moreover, cdG is a significant component of Complex 3 and participates in several essential pathways of cell cycle regulation in *C. crescentus*. For example, cdG binds to CckA and ShkA to induce phosphatase and kinase activity, respectively. While CckA controls the phosphorylation/dephosphorylation of several proteins, such as CtrA and CpdR, ShkA:cdG regulates the phosphorylation of TacA, which downregulates the stalked pole muramidase homolog SpmX and the stalk length regulator StaR [50]. Additionally, cdG has been verified to participate in the stress response, contributing to the survival of *Caulobacter* in oligotrophic environments [51]. Due to the importance of cdG, our protease complex model is potentially a valuable tool for understanding the regulatory network of *C. crescentus*.

With the advances in experimental technologies, mRNA and protein abundance of master regulators have been monitored and measured throughout the cell cycle. However, there is a limited comparison between experiments and simulations. Our results align very well with the experimental data. Satisfactory simulation results of our model, as indicated by visual inspection, suggest that the proposed regulatory network appropriately characterizes the *Caulobacter* cell cycle progression. This study also suggests the cell cycle dependent proteolysis of CtrA is significant for the cell cycle regulations and robustness. Our model can capture major features of seven mutant strains, which has the potential to predict phenotypes of nonviable mutant strains and functions of involved proteins. As most molecules involved in our model (CtrA, CcrM, GcrA, DnaA, etc.) are conserved among proteobacteria [18, 52, 53], this framework could be applied to the study of other proteobacteria. Last but not least, this work is a successful application of multiobjective optimization problem, showing that MOP is a promising approach for handling conflicting objectives in biological modeling.
